# Coherent quantum depletion of an interacting atom condensate

**DOI:** 10.1038/ncomms7624

**Published:** 2015-03-13

**Authors:** M. Kira

**Affiliations:** 1 Department of Physics, Philipps-University Marburg, Renthof 5, D-35032 Marburg, Germany

## Abstract

Sufficiently strong interactions promote coherent quantum transitions in spite of thermalization and losses, which are the adversaries of delicate effects such as reversibility and correlations. In atomic Bose–Einstein condensates (BECs), strong atom–atom interactions can eject atoms from the BEC to the normal component, yielding quantum depletion instead of temperature depletion. A recent experiment has already been verified to overcome losses. Here I show that it also achieves coherent quantum-depletion dynamics in a BEC swept fast enough from weak to strong atom–atom interactions. The elementary coherent process first excites the normal component into a liquid state that evolves into a spherical shell state, where the atom occupation peaks at a finite momentum to shield 50% of the BEC atoms from annihilation. The identified coherent processes resemble ultrafast semiconductor excitations expanding the scope of BEC explorations to many-body non-equilibrium studies.

## Abstract (web summary)

To overcome losses and thermalization, a quantum system requires strong interactions. Following recent experiments, Mackillo Kira shows that a BEC swept fast enough from weak to strong interactions exhibits coherent quantum-depletion dynamics dominated by particle clusters, resembling semiconductor excitations.

## Graphical abstract

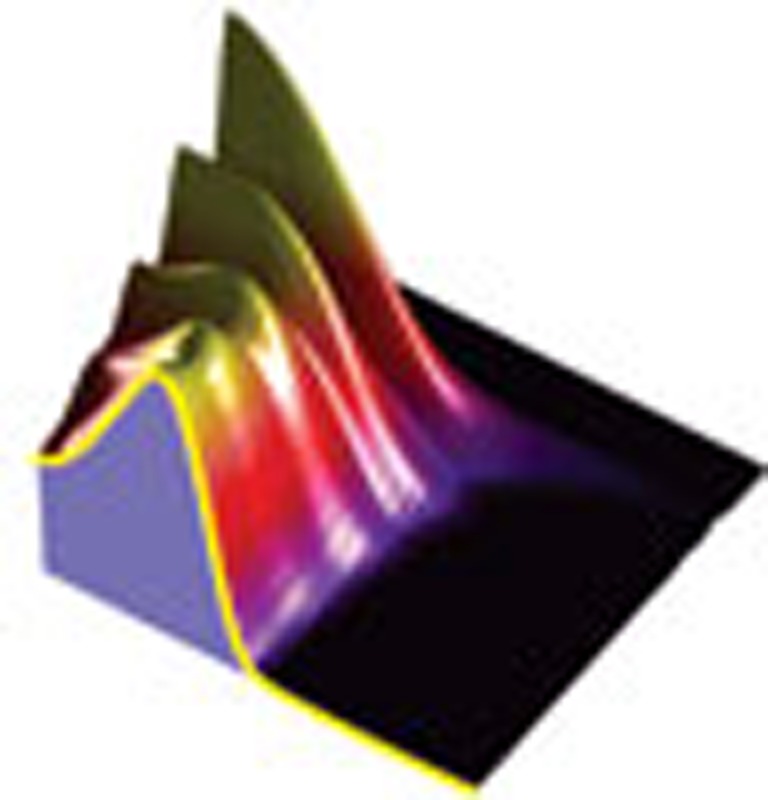

Strong interactions^1^,^2^,^3^ are prerequisite for efficient quantum-state manipulations hindered by dissipation and thermalization. For ultracold atoms, thermalization^4^ itself is advantageous because it creates the Bose–Einstein condensate (BEC) from the normal component, whereas quantum depletion^5^,^6^ ejects atoms from the BEC via interactions. However, coherent quantum depletion is reversible because it is driven by a distinct coherent transition amplitude between the BEC and normal-component atoms, unlike losses and thermal depletion^4^. Atom–atom interactions may also bind atoms to clusters within a range defined by the atomic scattering length^7^, *a*_scatt_, that can be substantially larger than the atom size; a large (small) |*a*_scatt_| implies strong (weak) interactions. The *a*_scatt_ can be systematically changed by applying an external magnetic field *B* to tune the atom coupling in the vicinity of a Feshbach resonance^8^. A fast *B* sweep can then jump the system from weak to strong interactions on a timescale much faster than the three-atom loss^9^ and thermalization. Figure 1 illustrates how an abrupt change in *a*_scatt_ changes atoms’ sphere of influence, with radius *a*_scatt_, from weak to strong interaction at time *t*=0. The strongest interaction is found at so-called unitarity^7^,^10^ where *a*_scatt_ diverges. Unitarity can be detrimental for the BEC because atoms can then scatter over large distances, as indicated by the overlapping spheres. Fast changes in *a*_scatt_ have been shown to induce, for example, Sakharov oscillations^11^ and Bogoliubov excitations^12^,^13^,^14^.

An important milestone was reached in the fast-sweep experiment by Makotyn *et al*.^15^ (f-s experiment) with a ^85^Rb BEC swept to unitarity in just 5 μs. Beating the expectations^16^,^17^, the BEC survived the sudden jump to unitarity by evolving into a new quasiequilibrium in roughly 100 μs, which was much faster than the three-body loss rate. Earlier analyses of the f-s experiment identified a two^18^- and three^19^-body contact in the high-momentum tail of an atom distribution, which explained^13^,^20^ the momentum-dependent relaxation of the distribution tail, and assigned^9^ the unexpectedly long lifetime of the BEC at unitarity to a long-lived three-body state. In addition, the possibility of observing coherent oscillations has been predicted^21^ in an interacting Bose gas. Using full many-body computations^22^,^23^,^24^,^25^, I show that the f-s experiment has also established another landmark—coherent quantum depletion dominated by atom-cluster excitations resembling ultrafast excitations in semiconductors^23^,^26^,^27^.

## Results

### Atom-cluster dynamics in quantum depletion

Conceptually, an abrupt change in *a*_scatt_ brings the BEC to a non-equilibrium in the same manner as an ultrafast laser excites solids^23^,^26^ or molecules^28^ to a non-equilibrium state. Ultrafast spectroscopy is renown for exciting both transient coherences^22^,^26^,^29^ and relaxation oscillations^27^ towards equilibrium. Therefore, one may expect that the f-s experiment accessed very similar effects. In semiconductors, ultrafast lasers can also excite a family of quasiparticles ranging from individual electrons and holes to correlated particle clusters^25^ such as the dropleton^30^. Analogously, it clearly is interesting to explore what kind of many-body states the f-s experiment has excited.

Solving fast-sweep generated excitations is challenging because a full quantum-kinetic description must resolve how interactions break up the BEC atoms, clustered to all orders^25^, into normal-component atoms that typically form independent clusters containing only few atoms. I apply a cluster-expansion approach^22^,^23^ in the excitation picture^25^. The BEC is then presented completely and exactly^25^ in terms of few atom clusters generated into the normal component; the approach is summarized in Supplementary Notes 1–4. Especially, both quantum-depletion and ultrafast semiconductor excitations share exactly the same cluster-dynamics structure where clusters are generated sequentially^23^,^24^ from small to large ones. I utilize this fundamental connection to systematically determine both the formally exact atom-cluster dynamics and a systematic truncation scheme, presented in Supplementary Note 1.

The coherence itself is identified by the transition amplitude 
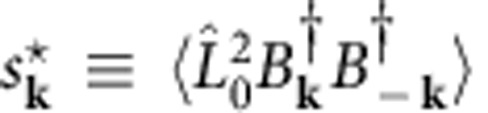
 to move two BEC atoms into the normal component. Microscopically, 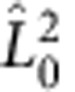 removes two atoms from the BEC and 
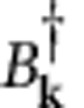
 is the usual boson-creation operator for a normal-component atom with a wave vector **k**≠0. For large atom traps, the momentum of the BEC atoms vanishes such that 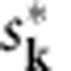 conserves the momentum by creating the atoms to momenta +*ħ***k** and −*ħ***k**. The accumulation of the normal component is determined by the atom occupation 
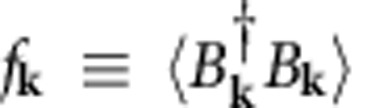
.

In the excitation picture^25^,^24^, *f*_**k**_ and *s*_**k**_ define the relevant two-atom clusters excited by the coherent quantum depletion. To focus the analysis on the possible coherent effects, I assume that the atom trap is closed, preventing thermalization because energy and particle exchange out of the trap is excluded. Consequently, the BEC atom number 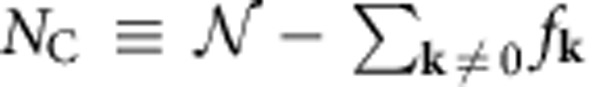 is the difference of total atom number 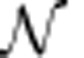 and the number of atoms in the normal component. The resulting (*s*_**k**_, *f*_**k**_, *N*_C_) group forms a closed set of two-particle cluster dynamics, constituting the hyperbolic Bloch equations (HBEs)^24^. As demonstrated in Supplementary Note 4, the HBEs describe gapless BEC excitations also at unitarity and the total atom distribution becomes


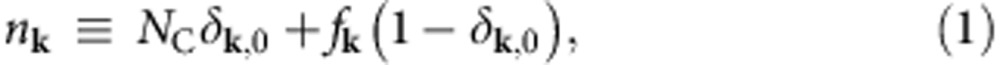


when no distinction is made between the BEC (**k**=0) and normal-component atoms.

### Connection of ultrafast semiconductor and BEC excitations

The HBEs resemble the semiconductor Bloch equations^27^,^31^ (SBEs) describing how ultrafast lasers drive coherent semiconductor polarization and the quantum kinetics^22^,^23^ that follows it, cf. Supplementary Note 2. In the HBEs, the coherent *s*_**k**_ is analogously driven by a *V*_**k**_ *N*_C_ source containing the Fourier transformation *V*_**k**_ of the pairwise atom–atom interaction. In analogy to the SBEs, the *V*_**k**_ *N*_C_ source is renormalized by a ∑_**k**′_*V*_**k**′−**k**_*s*_**k**′_ sum that describes the possibility of forming bound atom pairs called dimers^7^. Furthermore, *V*_**k**_ yields a hierarchical coupling to three- and four-atom-cluster dynamics, which introduces, for example, dephasing and energy shift to *s*_**k**_ (ref. 24). Three-atom-cluster dynamics also contains the possibility of forming Efimov trimers^24^,^32^.

Since atomic BECs and semiconductors are physically very different, it is clear that HBEs and SBEs yield also distinct differences. For example, the SBEs describe the dynamics of two kinds of fermions—electrons and holes—with different effective masses, which often produces different dynamics for the respective distributions; the HBEs used contain only one class of density distributions. The fermion character itself appears in the SBEs as Pauli blocking of transitions, contrasted by the bosonic stimulation in the HBEs. At the same time, *V*_**k**_ is a long-range interaction in the SBEs, whereas it has a short range in an atomic Bose gas. The long range and magnitude of *V*_**k**_ drives the relevant interaction timescales of the SBEs to be much faster (ultrafast sub-ps scale) than in the HBEs (μs scale).

In general, the semiconductors exhibit more possibilities of binding clusters due to stronger interactions and participation of more particles classes. Nevertheless, the cluster classes and phenomenology are often strikingly similar. For example, recently found dropletons^30^ emerge close to the pair-ionization threshold (corresponding to unitarity), have quantized energy and are spatially extended clusters with more than three electron—hole pairs. Similar to Efimov trimers, the dropleton is significantly larger (roughly 20 times) than a bound electron—hole pair, while its energy scaling is different.

Most important, the *s*_**k**_ drives coherent quantum depletion in the same way as the polarization embodies the coherences of the ultrafast semiconductor excitations. In semiconductors, the polarization determines the extent, symmetries and timescales of ultrafast excitations, whereas higher-order clusters, such as the dropletons, modify ‘only’ the quantitative details^33^. Since including additional hierarchical levels in the SBEs is numerically tedious, systematic and accurate methods have been developed to reduce it to few-parameter scattering models^22^ that satisfy the essential symmetries. Analogously, *s*_**k**_ dynamics must determine central aspects of the coherent quantum depletion, whereas the formation of, for example, Efimov trimers should yield ‘only’ quantitative modifications. When the hierarchical coupling is reduced to a dephasing *γ* and an energy-renormalization constant *μ* for *s*_**k**_, it must^24^ be accompanied by an excitation-dependent relaxation for the *f*_**k**_, based on symmetry; the explicit systematic model is presented in Supplementary Note 2.

The capabilities of this level HBEs is thoroughly tested by matching the computational parameters exactly with the conditions of the f-s experiment as given in ref. 15. The atom mass is that of ^85^Rb, the spherical trap has 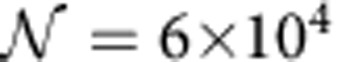 atoms and the average density is ‹*ρ*›=5.5 μm^−3^. The interaction is ramped from weak (*a*_scatt_=150 *a*_0_, *a*_0_ being the hydrogen Bohr radius) to unitarity (*a*_scatt_=∞) within 5 μs, which leaves *γ* and *μ* as the only free parameters chosen to match frequencies 0.761 and 0.475 kHz, respectively. The f-s experiment recorded the column distribution,


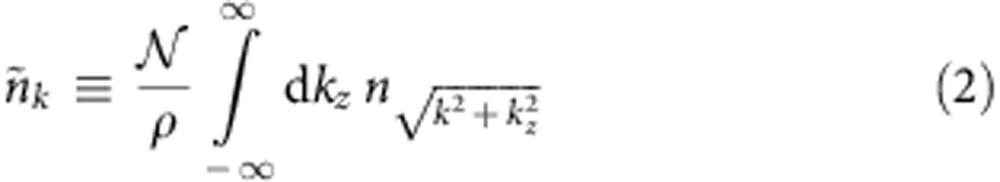


which is the marginal distribution of all atoms. In the f-s experiment, 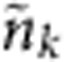 was broadened^15^ below *k*=3 μm^−1^ and included in the computations by using a broadened BEC distribution 
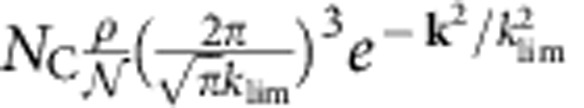
 to replace *N*_C_ *δ*_**k**,0_ in equation (1) with *k*_lim_=1.1342 μm^−1^.

### Quantum kinetics of quantum depletion

**Figure 2:**
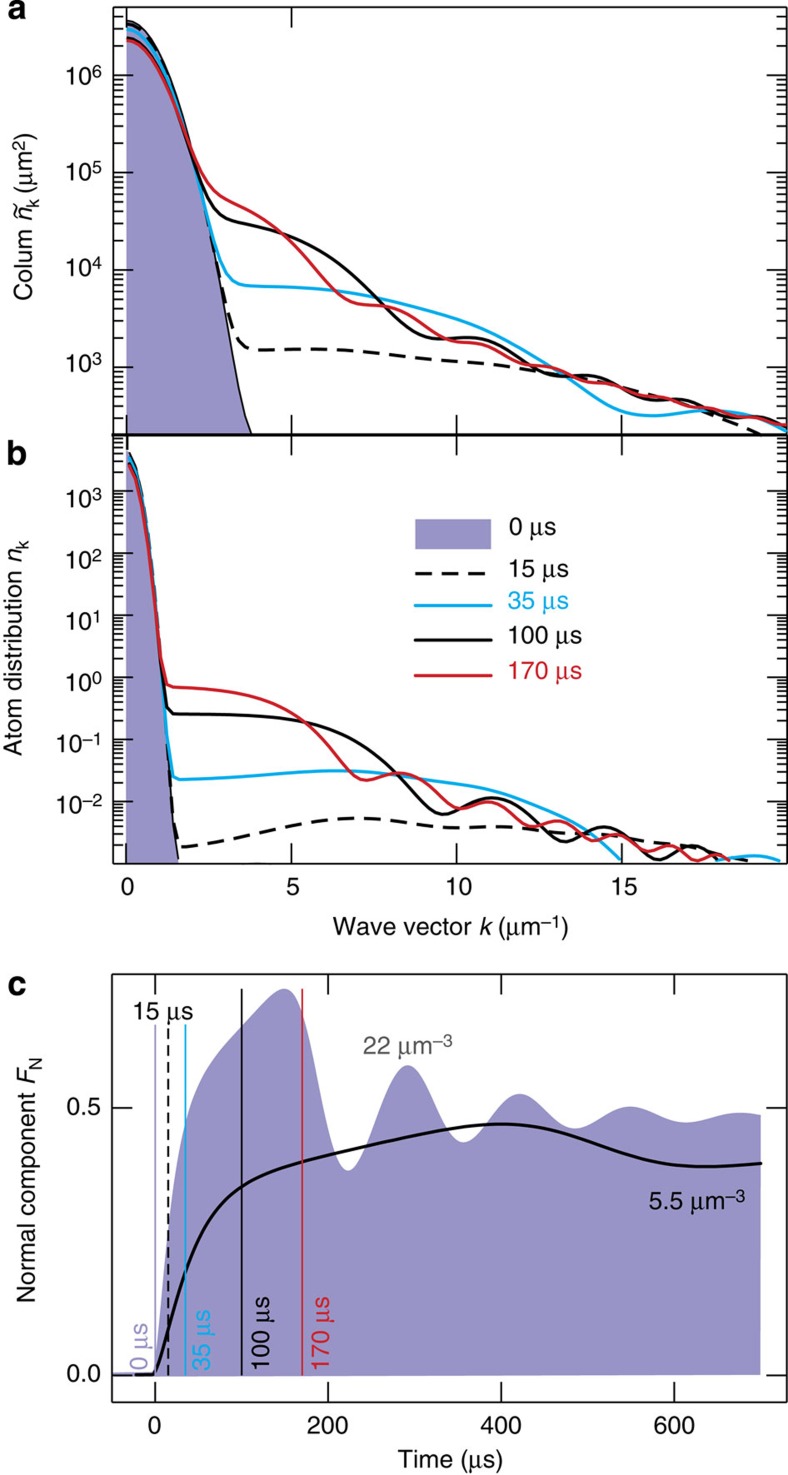
Fast-sweep analysis of interacting Bose gas. (**a**) Computed column distributions (2) for five representative *t*. (**b**) The corresponding actual *n*_**k**_ distributions. The interactions are switched to unitarity at *t*=0 and homogeneous ^85^Rb-atom density is *ρ*=5.5 μm^−3^. (**c**) Normal-component fraction for two representative *ρ*. The vertical lines identify the snapshots analysed in **a**,**b** with matching colour coding.

Figure 2a presents 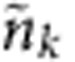 of the HBE computations for the same times as those recorded in the f-s experiment (Fig. 2 in ref. 15) where *t* is the evolution time at unitarity. At *t*=0 (shaded area), 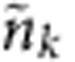 peaks at the origin because the system is still dominated by the BEC formed at the regime of weak interactions. Already *t*=15 μs evolution at unitarity (dashed line) produces an extended tail in 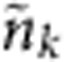. After that, the tail becomes steeper and evolves towards a steady state, while the BEC peak drops significantly. One also observes that the steady state is reached faster at high momenta (roughly above *k*=15 μm^−1^) while residual deviations remain in the vicinity of *k*=5 μm^−1^, even 170 μs after the switch is on. As shown in Supplementary Note 5, this computation explains quantitatively (*γ* and *μ* being the only free parameters) the f-s experiment timescales, overall excitation levels and distribution shapes, which confirms that the f-s experiment is indeed dominated by the coherent quantum depletion. To explain more quantitative details, one also needs to include the excitation-induced effects^22^,^33^,^34^,^35^,^36^,^37^ beyond a constant *μ*, as shown later.

The computed 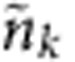 also exhibits slight oscillations for 100 μs (black line) and 170 μs (red line), which seem to be only indicative in the f-s experiment; this minor difference is probably related to the experimental resolution. The actual *n*_**k**_ distribution is plotted as in Fig. 2b using a very narrow *k*_lim_=0.3781 μm^−1^ for the BEC. A clear BEC peak is observed in *n*_**k**_ for all times, which shows that the BEC indeed exists even at unitarity, and that *n*_**k**_ and 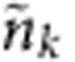 resemble each other. The projection in equation (2) simply smoothens the oscillatory features.

The level of quantum depletion is defined by the fraction of normal-component atoms 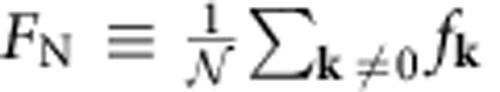, presented in Fig. 2b as function of time for *ρ*=5.5 μm^−3^ (line); the vertical lines identify the 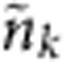 snapshots of Fig. 2a. For comparison, *F*_N_ is also shown for *ρ*=22 μm^−3^ (shaded area). As a general trend, *F*_N_ increases as *ρ* becomes larger. However, the coherent quantum depletion cannot completely exhaust the BEC (*N*_C_→0) because the *V*_**k**_ *N*_C_ source would become zero, which would reverse the coherent quantum depletion not sustained by its source. Instead, the steady-state *F*_N_ saturates towards 50% for large atom densities, for example, *ρ*=22 μm^−3^ yields pronounced relaxation oscillations towards 50% after peaking *F*_N_ to 72%. Also the *ρ*=5.5 μm^−3^ case shows some relaxation oscillations, but with a very long oscillation period.

**Figure 3:**
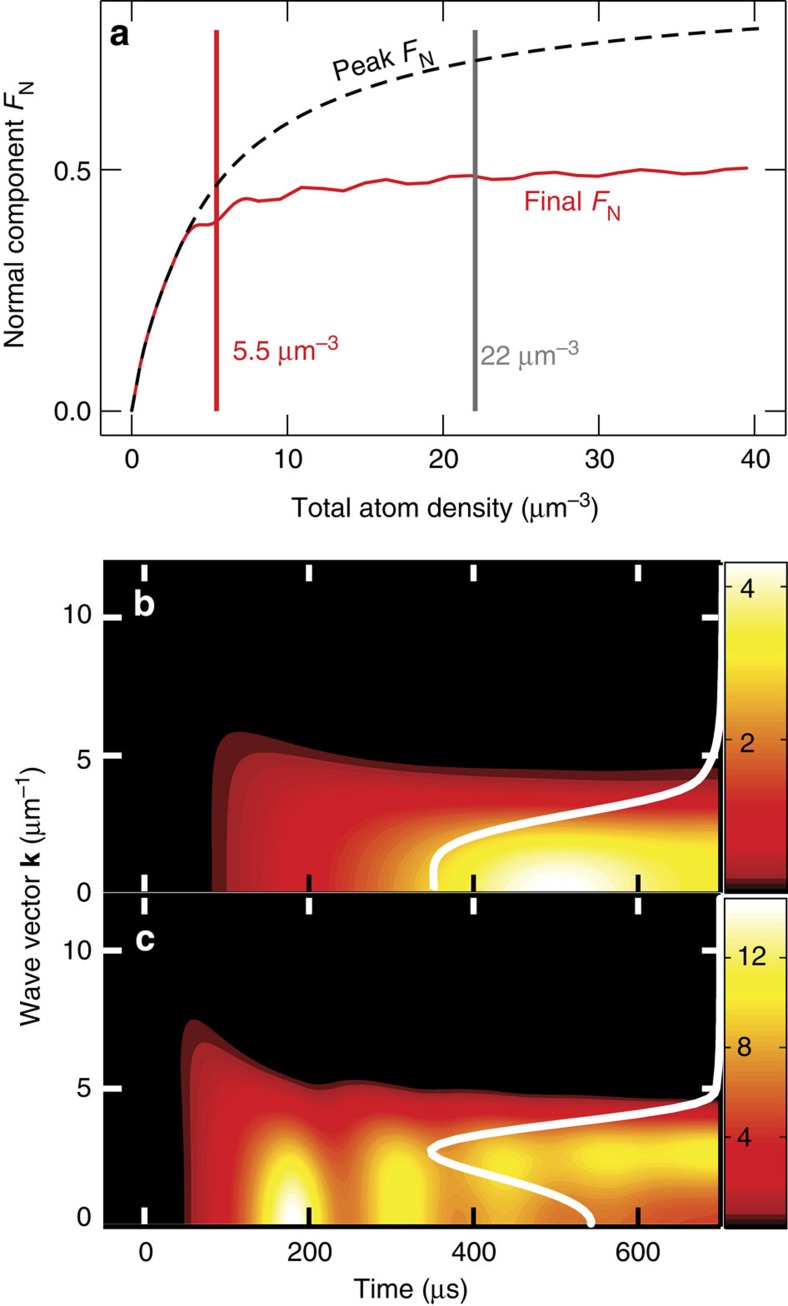
Shell-state formation in quantum depletion. (**a**) Peak (dashed line) and final (solid line, *t*=700 μs) fraction *F*_N_ of normal-component atoms generated by quantum depletion as function of density. The vertical lines indicate the densities analysed in **b**,**c** and Fig. 2. (**b**) Quantum kinetics of normal-component distribution for (**b**) *ρ*=5.5 μm^−3^ and (**c**) *ρ*=22 μm^−3^; the white line shows the shape of the final *f*_**k**_ at *t*=700 μs.

The *ρ* dependence of relaxation oscillations is studied in Fig. 3a by comparing the peak *F*_N_ (dashed line) with the final *F*_N_ (red-solid line) evaluated at *t*=700 μs; the vertical lines identify the densities of Fig. 2b. The final *F*_N_ saturates towards 50% from below at high densities, which shows that the coherent quantum depletion cannot collapse the BEC at the unitarity. For low densities, the peak and final *F*_N_ are identical while they deviate significantly above *ρ*=10 μm^−3^ due to the relaxation oscillations. Even though the f-s experiment does not show pronounced relaxation oscillations, they should become visible when *ρ* is increased by a factor of two.

### Shell-state formation

Quantum kinetics of normal-component *f*_**k**_ is presented in Fig. 3b,c as function of *t* for *ρ*=5.5 and 22 μm^−3^, respectively. The final distribution is indicated by the white line. Just after the switch is on (*t*=0), *f*_**k**_ is excited to large momenta and then focuses towards the origin, as in Fig. 2a. For *ρ*=5.5 μm^−3^, *f*_**k**_ evolves smoothly towards a monotonically decaying steady-state distribution. For *ρ*=22 μm^−3^, *f*_**k**_ dynamics does not only show the relaxation oscillations but it approaches an unexpected form; *f*_**k**_ peaks roughly at *k*=2.8 μm^−1^ and not at the origin. In other words, the normal-component atoms prefer to occupy a shell around the BEC due to the coherent *s*_**k**_; I will call this many-body configuration a shell state.

As validity checks, I show in Supplementary Note 4 that the shell state survives three-body loss, the excitation energies are indeed gapless and the results depend only slightly on the atom-trap density profile when the excitation-induced *μ*-shift moves the position of the shell state as function of density. Supplementary Note 3 also shows analytically that the shell state limits the coherently driven *F*_N_ to 50%. Therefore, the coherent quantum depletion alone cannot annihilate the BEC—one needs additional losses^38^,^39^ or thermalization to execute that. The unexpected survival of the BEC in the f-s experiment^15^ was assigned to build-up of long-living (1 ms) Efimov states^9^. The presented results also demonstrate that the BEC is additionally shielded by the shell state. The presence of the identified coherences is also expected to reduce the losses, as shown for semiconductors^33^.

**Figure 4:**
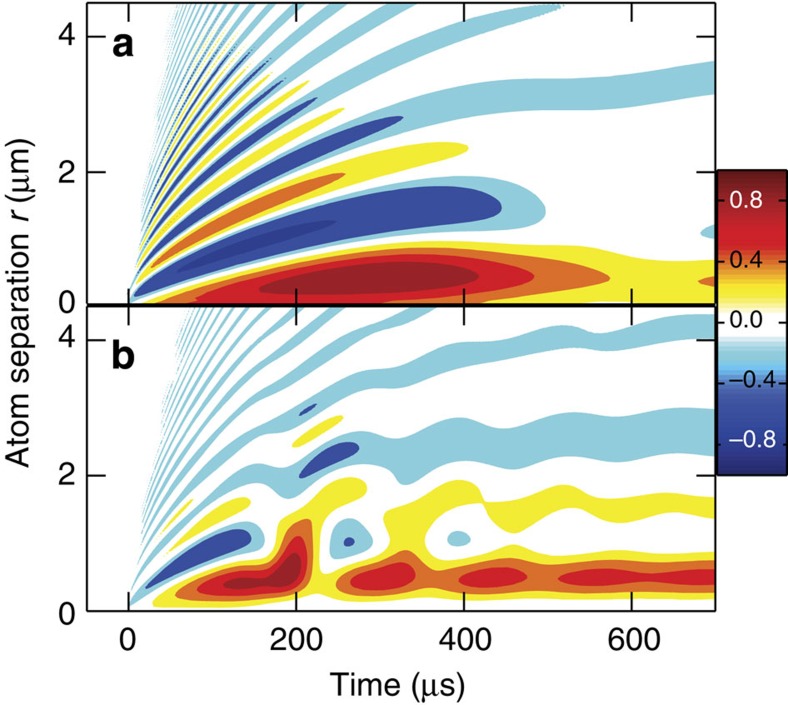
Long-range order in quantum depletion. Quantum kinetics of *r*^2^(*g*(**r**)−1) is shown for (**a**) *ρ*=5.5 μm^−3^ (peak *r*^2^(*g*(**r**)−1)=15.8 μm^2^) and (**b**) *ρ*=22 μm^−3^ (peak *r*^2^(*g*(**r**)−1)=22.3 μm^2^) as function of atom separation. The colour coding is scaled with respect to peak *r*^2^(*g*(**r**)−1).

The spatial extent of quantum depletion can be determined by analysing the atom–atom pair correlation function *g*(**r**) that defines the normalized probability of finding an atom at position **r** when another one is held at the origin. The normalization is chosen to produce *g*(**r**)=1 for uncorrelated atoms, and *r*^2^(*g*(**r**)−1) determines deviations with a radial weight, as discussed in Supplementary Note 3. Figure 4 shows the *r*^2^(*g*(**r**)−1) dynamics for *ρ*=5.5 μm^−3^ in Fig. 4a and *ρ*=22 μm^−3^ in Fig. 4b, studied also in Fig. 3b and Fig. 3c, respectively. Switching to unitarity at *t*=0 excites a long-range order extending beyond 4 μm and shows rapid oscillations that are typical for a liquid^40^ where atoms are arranged into shells. At later times, the atom–shell separation increases, indicated by the fan-like expansion of the peaks and dips. In addition, the height of large-distance shells diminishes leaving only one dominant peak and dip for *ρ*=5.5 μm^−3^, indicating transition to a short-range order. For *ρ*=22 μm^−3^, the steady-state *r*^2^(*g*(**r**)−1) has about three clear peaks and dips separated roughly by 2.1 μm matching well with the 2.2-μm wave length predicted to be the shell-state peak in *f*_**k**_. Therefore, the relaxation observed in Figs 2 and 3 is connected with coherent quantum kinetics from long-range-order liquid to a short-range-order configuration whose range can be extended by forming the shell state.

### Excitation-induced effects

**Figure 5:**
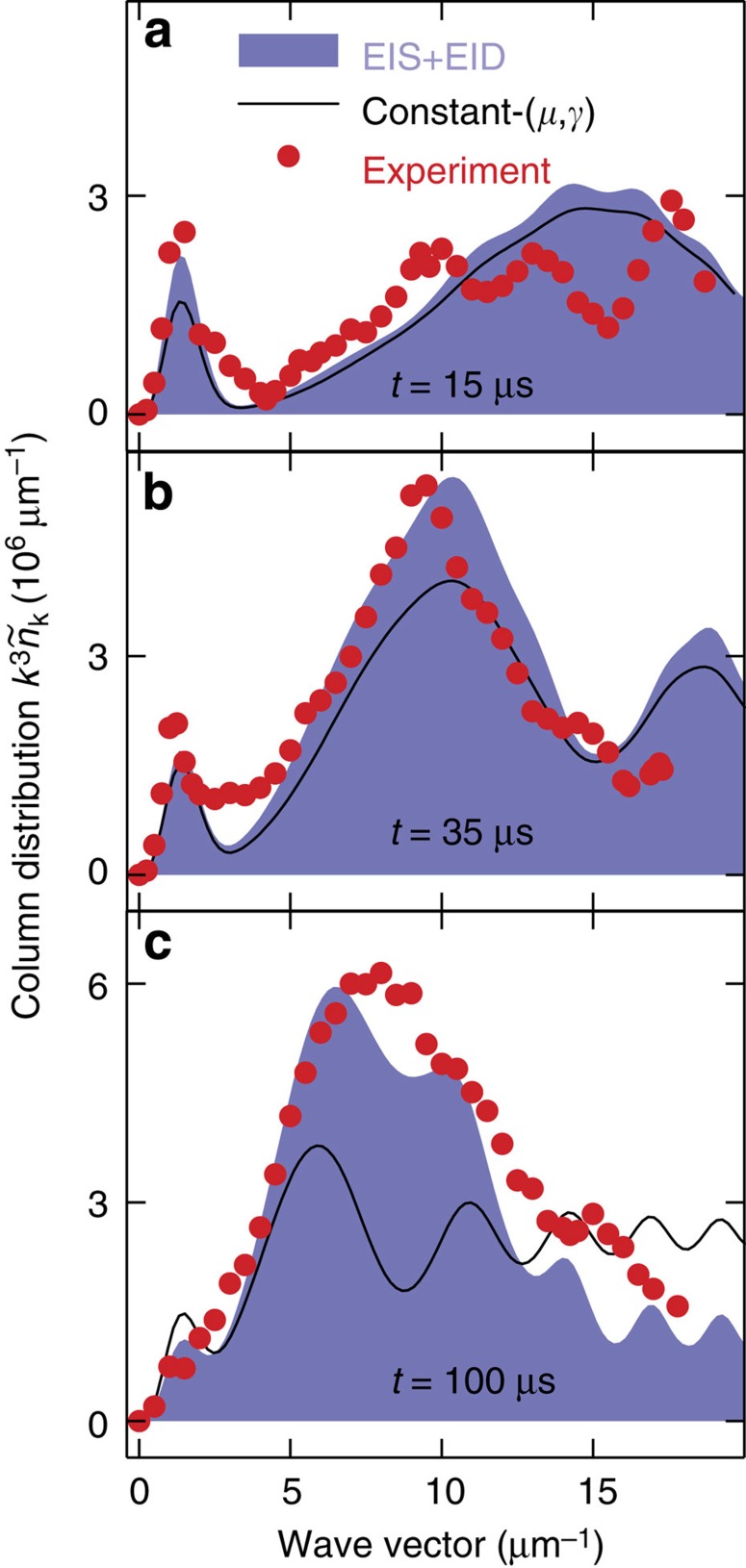
Quantitative experiment–theory comparison. Experimental (filled circles) 
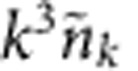
 is compared with constant-(*μ*, *γ*) (solid line) and full EIS/EID (shaded area) HBE computations for (**a**) *t*=15 μs, (**b**) *t*=35 μs and (**c**) *t*=100 μs after switching to unitarity. The experimental points are read from the inset to Fig. 2 in ref. 15.

To determine how quantitatively the computations already reproduce the f-s experiment, Fig. 5 compares 
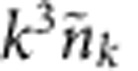
 of the f-s experiment (filled circles) with the constant-(*μ*,*γ*) computation (solid line). When the column distribution is multiplied by *k*^3^, the so-called two-body contact^19^,^41^,^42^ is observed as an asymptotically constant 
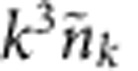
. A similar asymptotic behaviour is expected in the actual *k*^4^*n*_**k**_ distribution when multiplier *k*^4^ is used instead of *k*^3^ due to dimensionality difference of *n*_**k**_ and 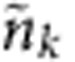.

Figure 5 shows that the constant-(*μ*,*γ*) computation already explains the experiment in great detail for the early times. However, it slightly underestimates *n*_*k*_ and 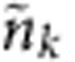 at late times, close to *k*=7 μm^−1^. Such quantitative differences are not unexpected because the excitation-induced shift (EIS) in *μ* and the excitation-induced dephasing (EID) in *γ* should modify the quantitative distribution/spectral shapes, as in semiconductors^33^.

To verify this explanation, I have also performed EIS computations that include the three-body loss, main features of EID/EIS and calculated the distributions using a local density approximation (LDA) average; details are given in Supplementary Note 5. Both EIS and EID are generated by the hierarchical coupling to three- and four-atom clusters that are induced by nonlinear source terms containing products of *f*_**k**_ and *s*_**k**_. As shown in ref. 24, such sources yield at least a quadratic density dependence for both *γ* and *μ*, in analogy to the three-body loss. To capture these excitation-induced aspects, I use the simplest functional form *μ*=211.4 Hz+628.9(*ρ*_n_/μm^−3^)^2^Hz and *γ*=21.14 Hz+10.48(*ρ*_n_/μm^−3^)^2^Hz, where *ρ*_n_ is the density of normal-component atoms. Many combinations of constant and quadratic coefficients produce qualitatively similar 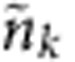 results; the used specific values yield roughly the best quantitative agreement with the experiment. The three-body loss model is defined in Supplementary Note 4 and its time constant is chosen to produce a 630-μs decay at ‹*ρ*›=5.5 μm^−3^, as in the experiment^15^. Owing to density dependence of the three-atom loss, *μ* and *γ*, the shape of 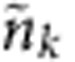 depends so strongly on the local density that the LDA average of Supplementary Note 4 needs to be implemented for a precise comparison.

The shaded areas in Fig. 5 present the LDA-averaged 
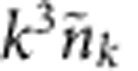
 of the EIS/EID computation. At early times (*t*=15 μs), the EIS/EID and constant-(*μ*, *γ*) computation (solid line) distributions are essentially the same, both explaining the experiment very well. However, only the EIS/EID computation agrees with the experiment quantitatively at *t*=100 μs. In other words, this analysis verifies that the quantitative deviations between the experiment and constant-(*μ*, *γ*) computation stem from the excitation-induced effects. Both computations explain the presence of weak oscillations in the experiment as relaxation oscillations. While the oscillation period agrees well, there are some differences in the experimental versus theoretical oscillation amplitude. However, the deviation is close to the experimental noise level such that one would need to measure these oscillations with a finer resolution and probably compute the excitation-induced effects fully quantum kinetically to explain all quantitative details; both studies are beyond the scope of the present investigation.

## Discussion

Quantum depletion can be partially described in terms of the Bogoliubov excitations^24^, but they alone destroy the BEC at high densities^13^ because one needs the shell state to shield the BEC. The HBEs include the Bogoliubov excitations as a substructure^24^, while the shell state as well as dimers result from the additional coupling among them. In fact, the coherent quantum depletion drives many dimer resonances, especially just after the jump to unitarity, based on the time–energy uncertainty. All of the initially generated dimer components in *s*_**k**_ evolve with their own eigen frequency, which generates quantum beats as the system evolves further, explaining the observed *n*_**k**_ oscillations and the transient long-range order. The relaxation oscillations appear through the nonlinear modulation in the quantum-depletion source.

The presented quantum-kinetic theory explains how an atomic BEC generates the normal component via sequential atom clustering when the atom–atom interactions are switched fast to unitarity. The resulting kinetics is initiated by a transition amplitude describing a coherent ejection of two BEC atoms to the normal component. This process is shown to explain the recent f-s experiment^15^ in great detail, which connects quantum depletion with coherent non-equilibrium processes in semiconductors.

The coherent quantum depletion identified should exhibit partial reversibility when switched back to weak interactions. Coherent control should also become possible when a sequence of Feshbach-resonance modulations is implemented. For atom densities that are roughly two times higher than in the Makotyn *et al*.^15^ experiment, the coherent quantum-depletion process is shown to form a shell state faster than 50 μs even when three-body losses are present, which prevents quantum depletion beyond 50% normal-component fraction at quasiequilibrium.

The identification of the shell state is simplest if one can experimentally access the normal-component distribution *f*_**k**_ of a homogeneous density. However, current measurements detect the overall distribution *n*_**k**_ including both the BEC and the normal component, and measure its density average. In a realistic experiment, the BEC contribution of *n*_**k**_ spreads around **k**=0 due to finite-size effects^15^. The visibility of the shell state can, therefore, be enhanced by decreasing the BEC spreading and/or by having a flatter atom trap, which makes the density profile more homogeneous. Therefore, further experiments and/or analyses are needed for an unambiguous identification of the shell state. The computations suggest that the shell state should become appreciable at atom densities that are roughly two times higher than in ref. 15, and that the excitation-induced effects can be accessed by comparing experiment and theory 
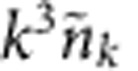
 distributions quantitatively; an analogous analysis has delivered very precise insights into the many-body states involved in semiconductor excitations^33^.

The predicted non-equilibrium dynamics as well as shell-state formation can be experimentally accessed by measuring the evolution of atom distribution *n*_**k**_, which also provides direct information of the normal-component fraction and its shape. I also expect that the effect of higher-order clusters (such as Efimov trimers) can be identified through quantitative shape changes in the *n*_**k**_, in analogy to the cluster-based studies in semiconductors^30^,^33^. Experiments with multiple switch-on/off sequences may access analogous coherent non-equilibrium quantum kinetics as in ultrafast semiconductor excitations. Through this connection, one may extend the future fast-sweep experiments to utilize novel concepts such as coherent control^43^ and quantum spectroscopy^30^,^44^,^45^ to access unexpected many-body states.

## Methods

### General aspects

Coherent quantum depletion is solved with the HBEs^24^ that establish a new method to study an interacting Bose gas. The HBEs are presented in Supplementary Note 1 while the actual cluster-expansion^22^,^23^ derivation is performed in ref. 24. The HBEs are analogous to the SBEs that govern the physics of ultrafast excitations in semiconductors^22^,^23^,^27^. Using the formal HBE–SBE connection, the quantum-kinetics insights of semiconductor studies are applied to reduce the hierarchical many-body coupling to a dephasing and energy shift that conserve the energy and maintain the BEC excitation as a minimum uncertainty state of the Heisenberg uncertainty relation. At this level, the method studies exclusively coherent quantum depletion because it deliberately omits atom loss, energy loss and thermalization. Atom losses can be included straightforwardly as explained in Supplementary Note 4.

### Numerical aspects

The HBEs couple the coherent transition amplitude *s*_**k**_ with the normal component and BEC occupations *f*_**k**_ and *N*_C_, respectively. Owing to the radial symmetry, one needs to consider only the radial *k* dependency for both *s*_**k**_ and *f*_**k**_. The resulting HBE computations are performed by discretizing *k* into 800 intervals and the atom–atom interactions are modelled with a realistic Morse potential. In order to resolve both the short- and long-range quantum kinetics of the interacting atoms, the *k* grid is divided into two parts: the low-*k* grid extends up to 76 μm^−1^ and the high-*k* grid reaches 6.0 × 10^4^ μm^−1^, both containing 400 points. This grid covers *k* values for over 5 orders of magnitude, which is sufficient to reveal how the atom–atom interactions exhibit repulsion <100 pm and unitarity interaction extending to macroscopic distances. The HBE dynamics is then solved with a fourth-order Runge–Kutta method.

## Author contributions

M.K. is solely responsible for the scientific content of this work.

## Additional information

**How to cite this article:** Kira, M. Coherent quantum depletion of an interacting atom condensate. *Nat. Commun.* 6:6624 doi: 10.1038/ncomms7624 (2015).

## Supplementary Material

Supplementary Information Supplementary Figures 1-6, Supplementary Notes 1-5 and Supplementary References

## Figures and Tables

**Figure 1:**
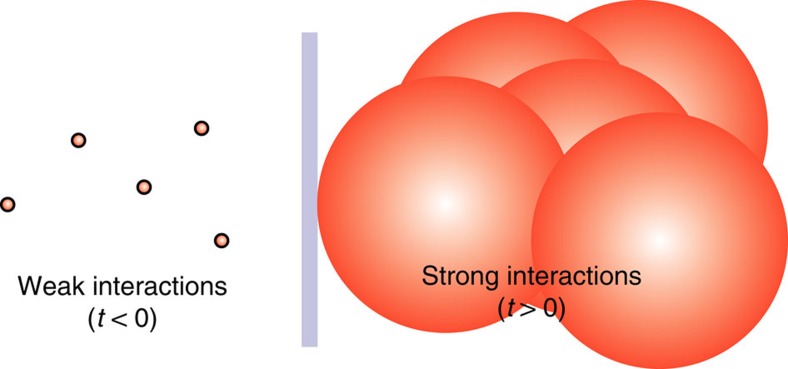
Principle of fast-sweep experiments. Schematic illustration of the range of *a*_scatt_ change in fast-sweep experiments switched at time *t*=0 from weak (left) to strong (right) interactions; the sphere of influence has radius *a*_scatt_ around each atom.
