# Supplementary material for: Coherent quantum depletion of an interacting atom condensate
Source: Nat Commun. 2015 Mar 13;6:6624. doi: 10.1038/ncomms7624 (PMC4382704; doi:10.1038/ncomms7624)
Supplement: Supplementary Information — Supplementary Figures 1-6, Supplementary Notes 1-5 and Supplementary References [file ncomms7624-s1.pdf]

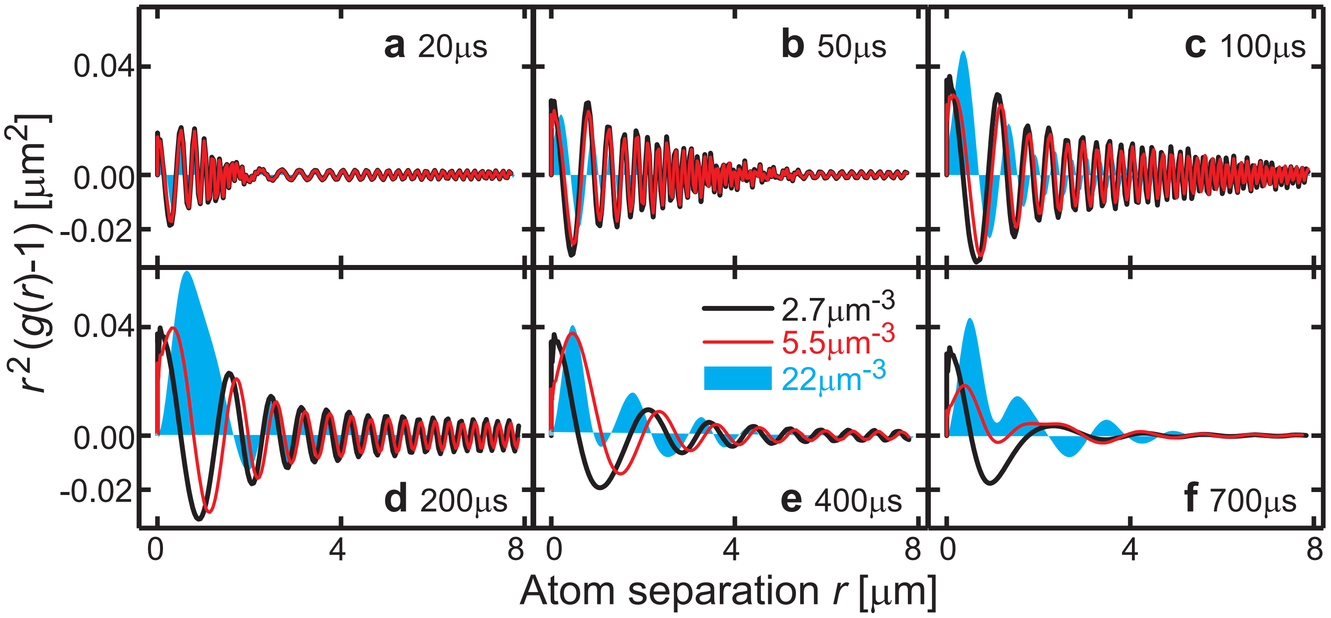

Supplementary Figure 1: **Computed atom–atom correlations at large length scales**. Correlation $r^2(g(r)-1)$ is plotted as function of r for density $\rho = 2.7/\mu\mathrm{m}^3$ (black line), $\rho = 5.5/\mu\mathrm{m}^3$ (red line), and $\rho = 22/\mu\mathrm{m}^3$ (shaded area). The BEC has evolved at unitarity for time t indicated in each frame.

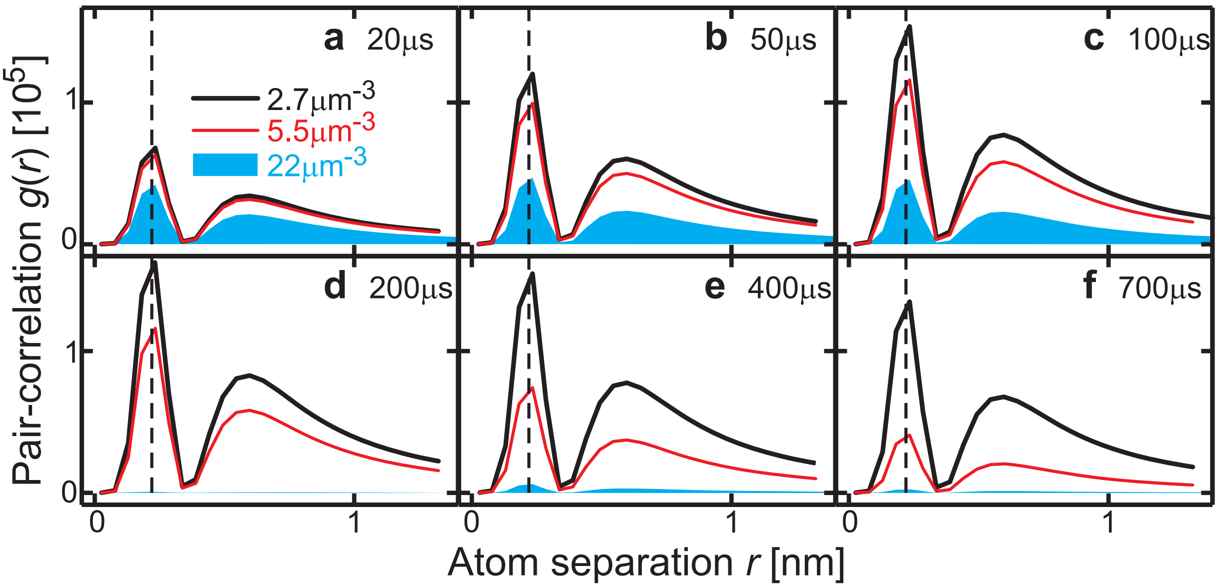

Supplementary Figure 2: **Computed atom–atom correlations at atomic length scales**. Correlation $g(r)$ as function of r; otherwise, computation, line styles, and selected times are as in Supplementary Figure 1.

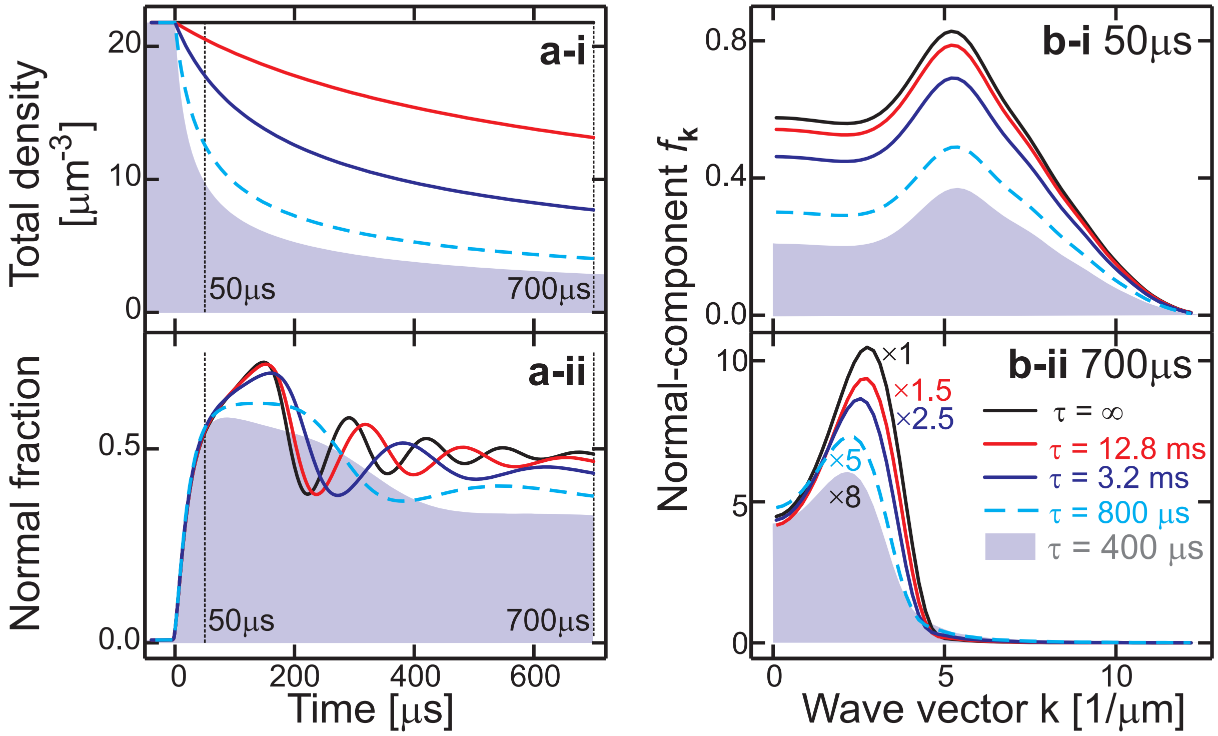

Supplementary Figure 3: **Quantum kinetics of the shell-state and the three-body loss**. The dynamics of **a-i** the total atom density vs. **a-ii** the normal-component fraction for infinite (black line), 12.8 ms (red line), 3.2 ms (blue line), and 800 μs (dashed line), and 400 μs three-body loss time at $\rho = 5.5\,\mu\mathrm{m}^3$; the initial density is $\rho = 22\,\mu\mathrm{m}^3$ and the vertical dotted lines indicate the snapshot times. The corresponding normal-component distributions after **b-i** 50 μs and **b-ii** 700 μs evolution at unitarity.

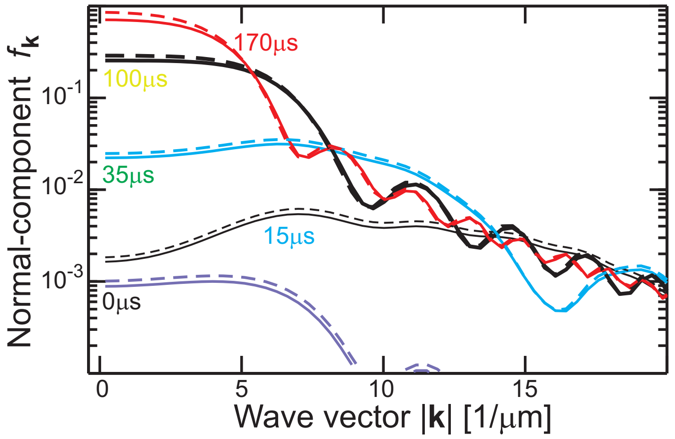

Supplementary Figure 4: **Effect of density profile on quantum depletion**. The normal-component distribution $f_{\mathbf{k}}$ as function of $|\mathbf{k}|$; selected times and color coding as in Fig. 2 of the main text. Solid lines are computed with a homogeneous density of $\rho = 5.5/\mu\mathrm{m}^3$ and the dashed lines are LDA results corresponding to the average density $\langle\rho\rangle = 5.5/\mu\mathrm{m}^3$.

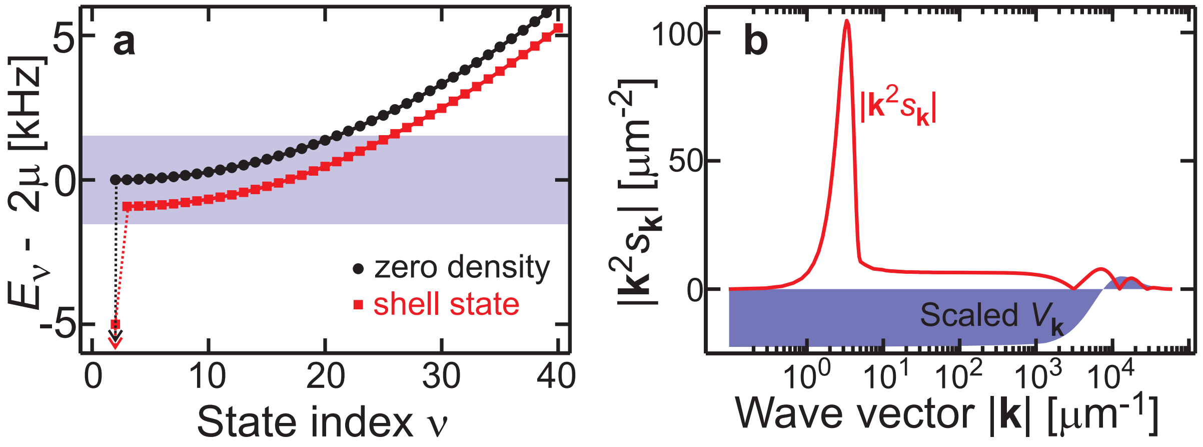

Supplementary Figure 5: **Gapless excitations of the HBEs**. **a** Computed excitation eigen states $(E_\nu - 2\mu)$ as function of state index for zero-density (black circles) vs. final shell state of Fig. 3**c** in the main text. The solid (dotted) line is a guide to the eye for the continuum (bound) states. The deeply bound state is found at $E_1 = -10.6$MHz (out of scale). The shaded area indicates the $\pm 2\gamma$ range of the resonant HBE excitations. **b** The Tan contact tail of $s_\mathbf{k}$ is analyzed by plotting $|\mathbf{k}^2 s_\mathbf{k}|$ (red line) as function of $\mathbf{k}$. The shaded area shows the corresponding $V_\mathbf{k}$ (scaled).

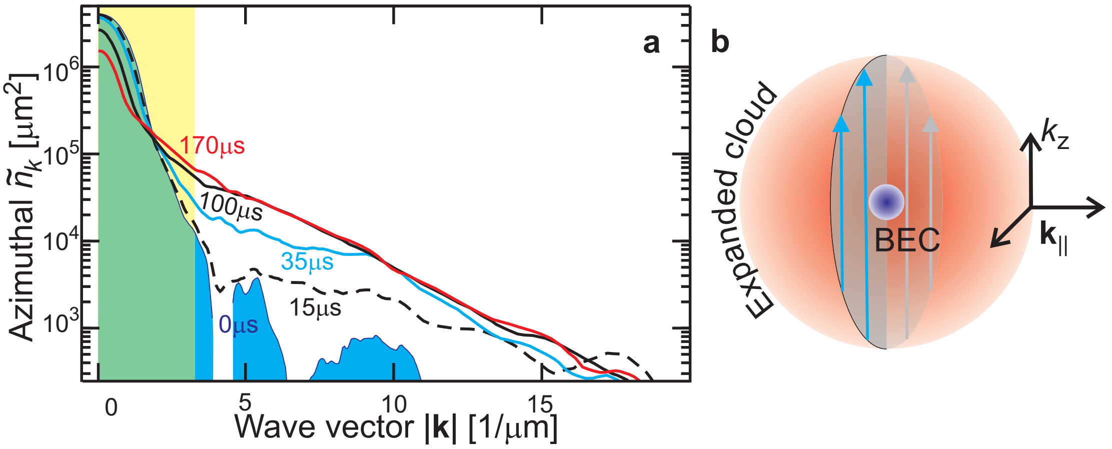

Supplementary Figure 6: **Measured $\tilde{n}_k$ dynamics at unitarity**. **a** The measured $\tilde{n}_k$ as function of $k = |\mathbf{k}_{\|}|$, read from Ref. 1. The normalization, selected evolution times, and color coding as in Fig. 2 in the main text. The transparent yellow area indicates the part of the data that is contaminated by the initial-size effects. **b** Imaging scheme of $\tilde{n}_k$. The blue (red) sphere symbolizes the BEC inside a trap (atom cloud after opening the trap). Arrows identify the direction of imaging beams counting the atoms.

Supplementary Note 1: Many-body theory of interacting Bose gas

Many-particle properties of bosonic atoms can be conveniently described by introducing the bosonic field operators $\hat{\Psi}(\mathbf{r})$ and $\hat{\Psi}^\dagger(\mathbf{r})$. Inside a trap, atoms experience a pair-wise interaction $V(\mathbf{r})$ besides the confinement potential $U(\mathbf{r})$. The resulting many-body Hamiltonian becomes then

$$\hat{H} = \int d^3r\, \hat{\Psi}^\dagger(\mathbf{r}) \left[-\frac{\hbar^2\nabla^2}{2m} + U(\mathbf{r}) \right] \hat{\Psi}(\mathbf{r}) + \frac{1}{2} \int d^3r \int d^3r'\, \hat{\Psi}^\dagger(\mathbf{r}) \hat{\Psi}^\dagger(\mathbf{r}') V(\mathbf{r}' - \mathbf{r}) \hat{\Psi}(\mathbf{r}') \hat{\Psi}(\mathbf{r}) \,, \tag{1}$$

where atoms have a mass m. For ultralow temperatures, atom–atom interactions are describable by the so-called s-wave scattering[2,3,4,5,6,7] which reduces $V(\mathbf{r}) = V(|\mathbf{r}|)$ to radial symmetry. For later reference,

$$V_\mathbf{q} \equiv \frac{1}{\mathcal{L}^3} \int d^3r\, V(|\mathbf{r}|)\, e^{-i\mathbf{q}\cdot\mathbf{r}} \tag{2}$$

is the Fourier transform of the pair-wise interaction, and $\mathcal{L}^3$ is the quantization volume.

We may divide the trap into cells that are large enough to contain the full many-body dynamics and small enough to treat particles as homogeneous inside each cell having a volume $\mathcal{L}^3$. Therefore, we use a plane-wave expansion:

$$\hat{\Psi}(\mathbf{r}) = \frac{1}{\mathcal{L}^{3/2}} \sum_{\mathbf{k}} e^{i\mathbf{k}\cdot\mathbf{r}}\, B_{\mathbf{k}}\,, \qquad \hat{\Psi}^\dagger(\mathbf{r}) = \frac{1}{\mathcal{L}^{3/2}} \sum_{\mathbf{k}} e^{-i\mathbf{k}\cdot\mathbf{r}}\, B^\dagger_{\mathbf{k}}\,, \tag{3}$$

where $B_{\mathbf{k}}$ and $B^\dagger_{\mathbf{k}}$ are the usual boson operators for atoms with the wave vector $\mathbf{k}$ and the momentum $\hbar\mathbf{k}$. The division into cells generalizes a purely homogeneous computation to include an arbitrary atom-density profile in the spirit of the local-density approximation (LDA), discussed in Supplementary Note 4.

Within each cell, the BEC is defined by a single boson operator that has a vanishing $\mathbf{k}$, whereas the $\mathbf{k} \neq 0$ bosons denote atoms in the normal component. Substituting the field operators (3) into Eq. (1) yields[8]

$$\begin{aligned}\hat{H} \;=\; & \sum_{\mathbf{k}}{}' \frac{\hbar^2\mathbf{k}^2}{2m}\, B^\dagger_{\mathbf{k}} B_{\mathbf{k}} + \frac{V_0}{2}\left(B^\dagger_0 B^\dagger_0 B_0\, B_0 + 2\sum_{\mathbf{k}}{}' B^\dagger_0 B^\dagger_{\mathbf{k}} B_{\mathbf{k}}\, B_0 + \sum_{\mathbf{k},\mathbf{k}'}{}' B^\dagger_{\mathbf{k}} B^\dagger_{\mathbf{k}'} B_{\mathbf{k}'}\, B_{\mathbf{k}} \right) \\ & + \sum_{\mathbf{k}}{}' V_{\mathbf{k}} B^\dagger_0 B^\dagger_{\mathbf{k}} B_{\mathbf{k}}\, B_0 + \frac{1}{2}\sum_{\mathbf{k}}{}' V_{\mathbf{k}} \left[B^\dagger_0 B^\dagger_0 B_{\mathbf{k}}\, B_{-\mathbf{k}} + B^\dagger_{-\mathbf{k}} B^\dagger_{\mathbf{k}} B_0 B_0 \right] \\ & + \sum_{\mathbf{k},\mathbf{k}'}{}' V_{\mathbf{k}} \left[B^\dagger_0 B^\dagger_{\mathbf{k}+\mathbf{k}'} B_{\mathbf{k}'}\, B_{\mathbf{k}} + B^\dagger_{\mathbf{k}}\, B^\dagger_{\mathbf{k}'} B_{\mathbf{k}+\mathbf{k}'} B_0 \right] + \frac{1}{2} \sum_{\mathbf{k}\neq\mathbf{k}'}{}' \sum_{\mathbf{q}\neq(\mathbf{k},\mathbf{k}')} V_{\mathbf{k}-\mathbf{k}'} B^\dagger_{\mathbf{k}} B^\dagger_{\mathbf{q}-\mathbf{k}} B_{\mathbf{q}-\mathbf{k}'}\, B_{\mathbf{k}'}\end{aligned} \tag{4}$$

for each cell. Since $U(\mathbf{r})$ is essentially constant within each cell, it produces only a constant energy shift which is set to zero, for simplicity. The prime in the sum indicates that the condensate index $\mathbf{k} = 0$ or $\mathbf{k}' = 0$ is removed from it to explicitly isolate the BEC from normal-component contributions in the Hamiltonian.

Excitation picture

Hamiltonian (4) is exact, but it is not the most convenient starting point for cluster-expansion-based analyses[9] because atoms inside the BEC cluster to all orders, as shown in Ref. [8]. Transformation to the excitation picture[8] solves this problem. As a starting point, the many-body aspects are described by assuming that each cell is microcanonical such that the sum of BEC and normal-component atoms produces a constant $\mathcal{N}$. This is a reasonable assumption when the trap is a closed system or the cells are large enough to sustain a constant $\mathcal{N}$ on the time scales relevant for the many-body processes. For a later reference,

$$\hat{N}_{\mathrm{N}} \equiv \textstyle\sum_{\mathbf{k}}{}' B^\dagger_{\mathbf{k}} B_{\mathbf{k}}\,, \qquad \hat{N}_{\mathrm{C}} = \mathcal{N} - \hat{N}_{\mathrm{N}} \tag{5}$$

determine the total number operator for the normal-component and BEC atoms, respectively.

In order to define the excitation picture smoothly, it is useful to introduce the BEC rising and lowering operators $\hat{L}^\dagger_0$ and $\hat{L}_0$, respectively. They change the BEC number states $|n\rangle_0$ with simpler relations than B_0 and $B^\dagger_0$ do:

$$\hat{L}^\dagger_0 |n\rangle_0 = |n+1\rangle_0\,, \qquad \hat{L}_0 |n+1\rangle_0 = |n\rangle_0\,, \qquad \hat{L}_0 |0\rangle_0 = 0\,, \tag{6}$$

where $|n\rangle_0$ contains exactly n BEC atoms. The excitation picture is defined by a nonunitary transformation[8]

$$\hat{O}_{\mathrm{ex}} = \hat{T}_{\mathrm{ex}}\, \hat{O}\, \hat{T}^\dagger_{\mathrm{ex}}\,, \qquad \hat{T}_{\mathrm{ex}} \equiv \left(\hat{L}_0\right)^{\hat{N}_{\mathrm{C}}}\,, \tag{7}$$

where $\hat{N}_{\mathrm{C}}$ can be expressed in terms of $\hat{N}_{\mathrm{N}}$ with the help of definition (5). Since $\hat{T}_{\mathrm{ex}}$ is nonunitary, especially $\hat{T}_{\mathrm{ex}} \hat{T}^\dagger_{\mathrm{ex}} \neq \mathbb{I}$, the properties of the excitation picture are nontrivial. They are explicitly worked out in Ref. [8], and I next summarize only the main consequences. Both the expectation values and their dynamics

$$\langle \hat{O} \rangle = \mathrm{Tr}\left[O_{\mathrm{ex}}\, \hat{\rho}_{\mathrm{ex}}\right] \equiv \langle \hat{O}_{\mathrm{ex}} \rangle_{\mathrm{ex}}\,, \qquad i\hbar \frac{\partial}{\partial t} \langle \hat{O}_{\mathrm{ex}} \rangle_{\mathrm{ex}} = \langle \left[\hat{O}_{\mathrm{ex}},\, \hat{H}_{\mathrm{ex}}\right]_{-} \rangle_{\mathrm{ex}}\,, \tag{8}$$

respectively, can be computed completely within the excitation picture. In other words, the excitation picture does not introduce formal complications to the quantum kinetics. Most important, the *excitation picture eliminates all*

nontrivial BEC-related clusters and *excitations* exist exclusively in the normal component because the BEC component of the transformed density matrix $\hat{\rho}_{\rm ex}$ becomes a particle vacuum. In the excitation picture, the onset of quantum depletion can excite clusters only sequentially[9,10,11], from lower to higher clusters, as shown in Ref. [11].

This very feature yields the possibility to describe the many-body dynamics of interacting Bose gases with the same systematic cluster-expansion approach[8,9,11,12] as the one used fot semiconductors. The cluster-expansion approach is not perturbative in the traditional sense because it is formally exact[8,9] and yields an exact structure where a higher-order cluster is formed sequentially from the lower-order ones. In other words, in situations where excitation generates only low-order clusters, the omission of any higher-order cluster is valid as long as it has not been sequentially built up; the coherent quantum depletion satisfies this general form[11]. At the same time, the influence of higher-order clusters can often be systematically approximated with a simpler, symmetry conserving, form. In particular, this yields a systematic truncation of the hierarchy, which significantly extends the validity range of calculations including only low-rank clusters that are driven by a source, as shown in Refs.[10,12]. The interacting Bose gas casts into this form[11], and I use this property in my computations; further connections are provided in the main text and Supplementary Note 2.

The general form of $\hat{H}_{\rm ex}$ is rather complicated, but only the average BEC number $N_{\rm C} = \langle \hat{N}_{\rm C}\rangle$ and its fluctuations are relevant for the quantum-depletion physics. As shown in Ref. [8], the corresponding system Hamiltonian becomes

$$\begin{aligned}\hat{H}_{\rm ex} \;=\; & \frac{V_0}{2}\mathcal{N}(\mathcal{N}-1) + \sum_{\mathbf{k}}{}' \tilde{E}_{\mathbf{k}} B^\dagger_{\mathbf{k}} B_{\mathbf{k}} + \sum_{\mathbf{k}\neq\mathbf{k}'}{}' \sum_{\mathbf{q}\neq(\mathbf{k},\mathbf{k}')} \frac{V_{\mathbf{k}-\mathbf{k}'}}{2} B^\dagger_{\mathbf{k}} B^\dagger_{\mathbf{q}-\mathbf{k}} B_{\mathbf{q}-\mathbf{k}'} B_{\mathbf{k}'} \\ & + \sum_{\mathbf{k}}{}' \frac{N_{\rm C} V_{\mathbf{k}}}{2}\left[B_{\mathbf{k}} B_{-\mathbf{k}} + B^\dagger_{-\mathbf{k}} B^\dagger_{\mathbf{k}}\right] + \sum_{\mathbf{k},\mathbf{k}'}{}' \sqrt{N_{\rm C}}\, V_{\mathbf{k}} \left[B^\dagger_{\mathbf{k}+\mathbf{k}'} B_{\mathbf{k}'} B_{\mathbf{k}} + B^\dagger_{\mathbf{k}} B^\dagger_{\mathbf{k}'} B_{\mathbf{k}+\mathbf{k}'}\right]\,, \end{aligned} \tag{9}$$

where the occupation of BEC and normal-component atoms are given by

$$N_{\rm C} \equiv \mathcal{N} - \textstyle\sum_{\mathbf{k}} f_{\mathbf{k}}\,, \qquad f_{\mathbf{k}} \equiv \langle B^\dagger_{\mathbf{k}} B_{\mathbf{k}}\rangle = \langle B^\dagger_{\mathbf{k}} B_{\mathbf{k}}\rangle_{\rm ex} = \Delta\langle B^\dagger_{\mathbf{k}} B_{\mathbf{k}}\rangle_{\rm ex}\,, \tag{10}$$

respectively. Notation, $\Delta\langle\cdots\rangle_{\rm ex}$ means that all factorizations to lower-order atom clusters are removed from $\langle\cdots\rangle_{\rm ex}$, based on the Wick's theorem.[13] For boson systems, each creation or annihilation operator increases the cluster number by one.[14] For homogeneous systems, single-atom clusters (singlets) vanish[11] making two-atom clusters (doublets) the lowest contributing clusters. In the excitation picture, the quantum depletion first generates *only* doublets whereas the higher-rank atom clusters can form only sequentially after the initial transients.

Within the $\hat{H}_{\rm ex}$, the interactions modify the kinetic energy through $N_{\rm C}\, V_{\mathbf{k}}$ as well as through the fluctuations of the BEC number, yielding the *renormalized kinetic energy*[8]

$$\tilde{E}_{\mathbf{k}} \equiv \frac{\hbar^2\mathbf{k}^2}{2m} + N_C\, V_{\mathbf{k}} - \sum_{\mathbf{k}}{}' V_{\mathbf{k}}\,\left(f_{\mathbf{k}} + {\rm Re}\left[s_{\mathbf{k}}\right]\right) - \frac{1}{\sqrt{N_{\rm C}}} \sum_{\mathbf{k},\mathbf{k}'}{}' V_{\mathbf{k}}\, {\rm Re}\left[T^{\mathbf{k}',\mathbf{k}}_{1,2}\right]\,, \tag{11}$$

where $N_C\, V_{\mathbf{k}}$ introduces renormalization due to BEC while the other parts stem from the BEC-number fluctuations. These terms include the transition amplitude

$$s^\star_{\mathbf{k}} \equiv \langle \hat{L}_0\, \hat{L}_0\, B^\dagger_{\mathbf{k}} B^\dagger_{-\mathbf{k}}\rangle = \langle B^\dagger_{\mathbf{k}} B^\dagger_{-\mathbf{k}}\rangle_{\rm ex} = \Delta\langle B^\dagger_{\mathbf{k}} B^\dagger_{-\mathbf{k}}\rangle_{\rm ex}\,, \tag{12}$$

for a process where two BEC atoms are converted to normal-component atoms. In the excitation picture, $s_{\mathbf{k}}$ describes a pairwise correlation in a transition where two atoms are created to momenta $+\hbar\mathbf{k}$ and $-\hbar\mathbf{k}$. Physically, $s_{\mathbf{k}}$ identifies the coherence amplitude of quantum depletion, and it is equivalent to the two-mode squeezing[11,15] amplitude.

The $(s_{\mathbf{k}},\, f_{\mathbf{k}})$ pairs define the doublets of the interacting Bose gas. The renormalization (11) involves also a three-atom cluster (triplet) $T^{\mathbf{k}',\mathbf{k}}_{1,2}$. For the sake of completeness, all triplets (T) and quadruplets (Q) are defined by

$$T^{\mathbf{k}',\mathbf{k}}_{1,2} \equiv \Delta\langle B^\dagger_{\mathbf{k}+\mathbf{k}'} B_{\mathbf{k}'} B_{\mathbf{k}}\rangle_{\rm ex}\,, \qquad T^{\mathbf{k}',\mathbf{k}}_{0,3} \equiv \Delta\langle B_{-\mathbf{k}-\mathbf{k}'} B_{\mathbf{k}'} B_{\mathbf{k}}\rangle_{\rm ex}\,, \tag{13}$$

$$Q^{\mathbf{q},\mathbf{k}',\mathbf{k}}_{2,2} \equiv \Delta\langle B^\dagger_{\mathbf{k}} B^\dagger_{\mathbf{k}'} B_{\mathbf{k}'+\mathbf{q}} B_{\mathbf{k}-\mathbf{q}}\rangle_{\rm ex}\,, \quad Q^{\mathbf{q},\mathbf{k}',\mathbf{k}}_{1,3} \equiv \Delta\langle B^\dagger_{\mathbf{k}+\mathbf{k}'+\mathbf{q}} B_{\mathbf{q}} B_{\mathbf{k}'} B_{\mathbf{k}}\rangle_{\rm ex}\,, \quad Q^{\mathbf{q},\mathbf{k}',\mathbf{k}}_{0,4} \equiv \Delta\langle B_{-\mathbf{k}-\mathbf{k}'-\mathbf{q}} B_{\mathbf{q}} B_{\mathbf{k}'} B_{\mathbf{k}}\rangle_{\rm ex}\,, \tag{14}$$

where the subscripts label the number of creation (first index) and annihilation (second index) operators. The triplets and quadruplets include, e.g., the possibility to form Efimov trimers and quadmers[11], respectively.

Hyperbolic Bloch equations (HBEs)

The dynamics of all clusters is derived in Ref. [11], yielding the hyperbolic Bloch equations (HBEs),

$$i\hbar\frac{\partial}{\partial t}s_{\mathbf{k}} = 2E^{\rm ren}_{\mathbf{k}}\,s_{\mathbf{k}} + (1+2f_{\mathbf{k}})\,\Delta^{\rm ren}_{\mathbf{k}} - 2i\gamma_{\mathbf{k}}\,, \tag{15}$$

$$\hbar\frac{\partial}{\partial t}f_{\mathbf{k}} = 2\,{\rm Im}\,[\Delta^{\rm ren}_{\mathbf{k}}\,s^{\star}_{\mathbf{k}}] - \Gamma_{\mathbf{k}}\,, \tag{16}$$

$$\langle [B_0^\dagger]^J B_0^K\rangle = \delta_{J,K}\,\langle\tfrac{\hat N_{\rm C}!}{(\hat N_{\rm C}-J)!}\rangle_{\rm ex} \qquad \Rightarrow \qquad \langle B_0^\dagger B_0\rangle = \langle \hat N_{\rm C}\rangle_{\rm ex} = \mathcal{N} - \textstyle\sum_{\mathbf{k}} f_{\mathbf{k}} \equiv N_{\rm C}\,, \tag{17}$$

among the doublets. The quantum statistics of the BEC can be expressed completely with the normal-component expectation values $\langle\frac{\hat N_{\rm C}!}{(\hat N_{\rm C}-J)!}\rangle_{\rm ex} \equiv \langle \hat N_{\rm C}(\hat N_{\rm C}-1)\cdots(\hat N_{\rm C}-J+1)\rangle_{\rm ex}$ and $N_{\rm C}$ is the BEC-atom number (doublet) that appears explicitly in the HBEs. The higher order clusters appear only implicitly in $\gamma_{\mathbf{k}}$ and $\Gamma_{\mathbf{k}}$, defined below. The *HBEs are structurally exact* because $\gamma_{\mathbf{k}}$ and $\Gamma_{\mathbf{k}}$ describe the hierarchy problem, without further modifications. To simplify the notation, also the $\mathbf{k}=0$ term is included in the sums because, e.g., f_0 and s_0 cannot produce a macroscopic contribution under a sum, unlike a BEC.

The HBEs contain the *renormalized transition energy* and *quantum-depletion source*

$$E^{\rm ren}_{\mathbf{k}} \equiv \tilde E_{\mathbf{k}} + \sum_{\mathbf{k}'} V_{\mathbf{k}'-\mathbf{k}}\,f_{\mathbf{k}'}\,, \qquad \Delta^{\rm ren}_{\mathbf{k}} \equiv N_{\rm C}V_{\mathbf{k}} + \sum_{\mathbf{k}'} V_{\mathbf{k}'-\mathbf{k}}\,s_{\mathbf{k}'}\,, \tag{18}$$

respectively. Whenever BEC occupation and atom–atom interactions exist, the $N_{\rm C}V_{\mathbf{k}}$ part of the $\Delta^{\rm ren}_{\mathbf{k}}$ *directly* excites $s_{\mathbf{k}}$, initiating the quantum depletion. In other words, the $N_{\rm C}V_{\mathbf{k}}$ contribution is the only true source term for the quantum depletion, and $s_{\mathbf{k}}$ *assigns the coherent transient driven in fast-sweep experiments*. Once $s_{\mathbf{k}}$ becomes generated, the $\sum_{\mathbf{k}'} V_{\mathbf{k}'-\mathbf{k}}\,s_{\mathbf{k}'}$ renormalization modifies the quantum-depletion source, producing dimers as discussed in Supplementary Note 3. The HBEs also contain a hierarchical coupling to the triplets and quadruplets

$$2\gamma_{\mathbf{k}} = i\sqrt{N_{\rm C}}\sum_{\mathbf{l}}\left[V_{\mathbf{l}}\left(T^{\mathbf{l},-\mathbf{k}}_{0,3} + T^{\mathbf{l},\mathbf{k}}_{0,3}\right) + V_{\mathbf{l},\mathbf{k}}\left(T^{\mathbf{k}-\mathbf{l},-\mathbf{k}}_{1,2} + T^{\mathbf{l}-\mathbf{k},\mathbf{k}}_{1,2}\right)\right] + i\sum_{\mathbf{k}',\mathbf{q}} V_{\mathbf{q}}\left[Q^{\mathbf{k}'+\mathbf{q},\mathbf{k}-\mathbf{q},-\mathbf{q}}_{1,3} + Q^{\mathbf{q}-\mathbf{k}',-\mathbf{k}-\mathbf{q},\mathbf{k}}_{1,3}\right]\,, \tag{19}$$

$$\Gamma_{\mathbf{k}} = -2\,{\rm Im}\left[\sqrt{N_{\rm C}}\sum_{\mathbf{l}}\left(V_{\mathbf{l}}\,T^{\mathbf{l},\mathbf{l}-\mathbf{k}}_{1,2} - V_{\mathbf{l},\mathbf{k}}\,T^{\mathbf{l},\mathbf{k}}_{1,2}\right) + \sum_{\mathbf{k}',\mathbf{q}} V_{\mathbf{q}}\,Q^{\mathbf{q},\mathbf{k}',\mathbf{k}}_{2,2}\right]\,, \tag{20}$$

with $V_{\mathbf{l},\mathbf{k}} \equiv V_{\mathbf{l}} + V_{\mathbf{k}}$. These, e.g., create dephasing and additional energy renormalization for the doublets[12].

The HBE computations are performed with material parameters matching the analyzed experiment; the atom mass is set according to ^{85}Rb atoms that have a mass[16] $m = 84.9118\,u$ where u is the standard atomic weight. The individual ^{85}Rb atoms have a covalent radius[17] $r_0 = 220\,{\rm pm}$ that defines the typical length scale of atomic bonding, see Supplementary Figure 2. The corresponding pairwise atom–atom interactions are approximated by using the Morse potential[18] $V(\mathbf{r})$ that includes both the short-range atom–atom repulsion and the fast-decaying attractive part mimicking the van der Waals force. The attractive $V(\mathbf{r})$ dip is chosen to match the covalent radius. The Fourier-transformed $V_{\mathbf{k}}$ as well as the corresponding dimer scattering length $a_{\rm scatt}$ is derived in Ref. [11]. The amplitude of $V_{\mathbf{k}}$ is changed within $5\,\mu$s to sweep the system from $a_{\rm scatt} = 150\,a_0$ to $a_{\rm scatt} = \infty$, as in the experiment; this requires only a 1.7% change in the $V_{\mathbf{k}}$ amplitude; for more details on the Morse potential used, see Ref. [11] and Supplementary Figure 5**b**.

Supplementary Note 2: On the validity of the HBEs

As shown in Ref. 11, the HBEs include the Hartree-Fock-Bogoliubov (HFB) equations[19,20,21,22,23,24,25] as a subset, while introducing distinct differences that become decisive for a strongly interacting Bose gas. At the same time, Ref. 26 shows that the HFB approach yields an energy gap when the chemical potential is consistently determined only within the HFB approximation. If the excitation gap were also present in the HBEs, at least the long-time limit would be compromised because the BEC could not resonantly excite the normal component over the gap, diminishing the efficiency of the quantum depletion. However, even the HFBs describe correct gapless excitations if the chemical potential is replaced by a more general expression[26], which is of course ad hoc when one relies only on the HFB approximation. I will next discuss how the HBEs implement the shift of the chemical potential needed to systematically describe proper gapless excitation even under unitarity conditions.

Gapless excitations in HBEs

Despite the HBEs (15)–(17) being formally exact, the quality of the HBE solutions depends on how well the triplet/quadruplet contributions can be included in the doublet dynamics. In this paper, I have used a high-quality approximation identified below. To determine whether the corresponding HBE solution describes gapless excitations, it is useful to briefly review the differences of the HBE and HFB approaches.

The HBEs generalize the HFBs in multiple ways; (i) the Gross-Pitaevskii equation of the HFB approach is replaced by the BEC-statistics transformation (17), (ii) the HBEs contain two new energy renormalizations in Eq. (11):

$$\delta\mu \equiv -\sum_{\mathbf{k}}{}' V_{\mathbf{k}} \,\mathrm{Re}\left[f_{\mathbf{k}} + s_{\mathbf{k}}\right] , \qquad \delta\mu_T \equiv -\frac{1}{\sqrt{N_{\mathrm{C}}}} \sum_{\mathbf{k},\mathbf{k}'}{}' V_{\mathbf{k}} \,\mathrm{Re}\left[T_{1,2}^{\mathbf{k}',\mathbf{k}}\right] , \tag{21}$$

stemming from the number fluctuations of the BEC[8,11]. (iii) Additionally, the HBEs contain the hierarchical coupling to the triplets and quadruplets through the $\gamma_{\mathbf{k}}$ and $\Gamma_{\mathbf{k}}$ contributions given exactly by Eqs. (19)–(20). Altogether, the energy renormalization (11) and the imaginary part of $\gamma_{\mathbf{k}}$ produce an excitation-dependent shift (of the chemical potential) while $\Gamma_{\mathbf{k}}$ and the real part of $\gamma_{\mathbf{k}}$ result in a decay of the doublets. These effects are often referred to as the excitation-induced shift[27] and excitation-induced dephasing[28] in semiconductors. The energy-renormalization/dephasing physics is necessarily also present in a strongly interacting Bose gas because it contains the same cluster-dynamics structure as the semiconductors, discussed further below.

The explicit analysis of Supplementary Note 4 shows that these systematic shifts altogether produce gapless HBE excitations. Consequently, the cluster expansion inevitably yields gapless excitations, which follow only via an ad hoc implementation for the pure HFB equations. For infinitesimal excitations, the HFB gap as well as the cluster-expansion shifts become infinitesimal, making the HBE and HFB approaches identical for infinitesimally weak quantum-depletion levels.

Decay in HBEs

On a more fundamental level, both interacting Bose gases and semiconductors share the same sequential cluster-formation dynamics[9,11], and both systems are capable of producing similar highly correlated clusters with intriguing quantum phenomena. As the major advantage of the cluster expansion, a minimal amount of clusters determines the related many-body properties extremely efficiently[9] before the next level of clusters is generated. Therefore, one can use very similar strategies to solve the semiconductor and BEC problems, which may seem surprising, but follows from the general structure of the cluster dynamics. Despite being seemingly different, e.g., the Efimov and dropleton physics are highly analogous in the cluster-expansion sense, as discussed in the main text.

As a general tendency, formation of stable clusters tends to make formation of further clusters slow because the increased cluster size and binding makes inelastic cluster-forming collisions slower and less efficient. The exact time window of existence for a given cluster depends, naturally, on the system details. In principle, one can then use a maximum number of clusters in computations to follow how each cluster emerges, as has been done in simple systems[10]. Such a brute-force approach is neither meaningful nor feasible in strongly interacting Bose gas because the quantum-depletion aspects of doublets are still being charted while the numerical effort beyond this is very demanding. Judging from the state-of-the art in semiconductor computations[9,12], I estimate that the interacting Bose gas could be solved at the triplet-quadruplet level in a couple of years.

At the same time, the semiconductor studies[9] teach us that the hierarchical coupling, producing sophisticated quantum-cluster effects, can often be approximated with few parameters. More specifically, the semiconductor studies show[12] that a high-quality approximation must satisfy the symmetry structure of the hierarchical coupling. For example, the full form of the excitation-induced dephasing and shifts is very complicated while its diffusive structure is simple. Therefore, one can approximate it with a diffusive model[12] that contains only two parameters and still capture the main aspects of the excitation-induced dehasing and shifts. For example, the diffusive model replaces a Lorentzian absorption line by the correct asymmetric form, and even major excitation-induced shifts are well reproduced up to the ionization where the systems potentially contains dropletons.

In the current work, I have adopted an analogous reduction of the hierarchy by introducing dephasing and energy shifts that satisfies the central symmetries of triplets and quadruplets. As shown in Ref. 9, the doublets uniquely define quadrature fluctuations for each atomic Boson mode $\mathbf{k}$ that satisfies

$$\left(f_{\mathbf{k}} + \tfrac{1}{2}\right)^2 - |s_{\mathbf{k}}|^2 \geq \tfrac{1}{4} , \tag{22}$$

corresponding to the Heisenberg uncertainty relation. The equality is related to vanishing entropy and defines a minimum uncertainty state. The inequality implies that the system has thermal fluctuations. At low enough temperatures,

thermal fluctuations vanish such that the triplet/quadruplet scattering must maintain the system as a minimum-uncertainty state. Even though the exact triplet-quadruplet structure is complicated, its minimum-uncertainty conserving property is simple. We utilize this in order to produce a high-quality reduction of the full scattering, in analogy to the diffusive scattering model.

As the simplest model, $\gamma_{\mathbf{k}}$ induces dephasing $(\gamma - i\mu)\, s_{\mathbf{k}}$ for the coherent transition. By demanding that the HBE excitation remains a minumum-uncertainty state (Eq. (22) with an equal sign), $\Gamma_{\mathbf{k}}$ becomes excitation dependent

$$\gamma_{\mathbf{k}} = (\gamma - i\mu)\, s_{\mathbf{k}}\,, \qquad \Gamma_{\mathbf{k}} = \gamma \frac{2+2f_{\mathbf{k}}}{1+2f_{\mathbf{k}}} f_{\mathbf{k}}\,. \tag{23}$$

As demonstrated in Ref. [11], the dephasing choice (23) conserves entropy, energy, and the minimum-uncertainty state (equality in Eq. (22)), which is to be expected for a low-temperature Bose gas. As for semiconductors, the pragmatic validation of limits for this model can be judged either from experiments or from a higher-level cluster computation. Since the presented results indeed reproduce the experimental observations, as shown by comparison in Fig. 5 in the main text, the reduced dephasing model (23) is adequate for the analysis of this f-s experiment.

Strongly interacting Bose gases vs. semiconductors

In semiconductors, an elementary excitation process moves an electron from a conduction to a valence band, leaving an electronic vacancy, i.e. a hole, behind.[9] A laser pulse can efficiently generate such transitions via dipole interaction. For many illustrative examples, cf. Ref. [9] that investigates GaAs-type direct-gap semiconductor excitations involving one conduction and one valence band with a bandgap E_g between them. The semiconductor Bloch equations (SBEs)[29,30] establish the most accurate approach[31,32,33] to describe nonequilibrium many-body quantum kinetics of semiconductors excited by an ultrafast laser pulse.

To connect the HBEs with the SBEs, I will summarize quantum-well (QW) excitations for a situation where a laser pulse excites a large number of electrons and holes to a QW. Since electrons and holes are charged particles, they interact via Coulomb interaction. For the sake of comparison, I denote many of the quantities in the HBEs and SBEs with the same symbol, e.g., the pairwise Coulomb interaction is $V_{\mathbf{k}}$ even though it does not refer to an atom–atom interaction for the SBEs. In case of semiconductors (atoms), $V(\mathbf{r})$ has a long (short) range.

For the two-band system summarized here, the principal form of the SBEs follows from

$$\begin{aligned} i\hbar \frac{\partial}{\partial t} P_{\mathbf{k}} &= E^{\rm ren}_{\mathbf{k}}\, P_{\mathbf{k}} - \left(1 - f^e_{\mathbf{k}} - f^h_{\mathbf{k}}\right) \Delta^{\rm ren}_{\mathbf{k}} - i\gamma_{\mathbf{k}}\,, \\ \hbar \frac{\partial}{\partial t} f^\lambda_{\mathbf{k}} &= 2\,{\rm Im}\,[\Delta^{\rm ren}_{\mathbf{k}}\, P^\star_{\mathbf{k}}] - \Gamma^\lambda_{\mathbf{k}}\,, \qquad \lambda = e,\, h\,, \end{aligned} \tag{24}$$

where $P_{\mathbf{k}}$ is the microscopic polarization and $f^\lambda_{\mathbf{k}}$ is the electron ($\lambda = e$) or hole ($\lambda = h$) occupation excited by the electric field $E(t)$ of the laser pulse; a cluster-expansion-based derivation of the SBEs is presented, e.g. in Refs. [9,12]. The kinetic energy of the electron–hole pair becomes renormalized by the Coulomb interaction

$$E^{\rm ren}_{\mathbf{k}} \equiv E^{\rm eh}_{\mathbf{k}} - \sum_{\mathbf{k}'}{}' V_{\mathbf{k}'} \left(f^e_{\mathbf{k}'} + f^h_{\mathbf{k}'}\right)\,, \tag{25}$$

where the electron–hole pair energy $E^{\rm eh}_{\mathbf{k}} = \frac{\hbar^2 \mathbf{k}^2}{2m_{\rm r}} + E_g$ contains the reduced electron–hole mass $m_{\rm r}$; the quadratic dispersion is valid near the band extrema. The laser pulse appears in the SBEs through the renormalized Rabi energy

$$\Delta^{\rm ren}_{\mathbf{k}} \equiv d_{\rm eh}\, E(t) + \sum_{\mathbf{k}'} V_{\mathbf{k}'-\mathbf{k}}\, P_{\mathbf{k}'}\,, \tag{26}$$

where $E(t)$ is the electric field at the QW and $d_{\rm eh}$ is the dipole-matrix element for an electron–hole transition.

Equations (18) and (25)–(26) have the same structure — only the sign of density renormalizations is changed. Additionally, the polarization is directly driven as long as $E(t)$ is present at the QW whereas the normal component $s_{\mathbf{k}}$ is driven by the presence of the BEC. In other words, $E(t)$ and $N_{\rm C}$ are equivalent sources whereas $P_{\mathbf{k}}$ and $s_{\mathbf{k}}$ are equivalent coherent amplitudes excited by them, respectively. The electric field is treated self-consistently by solving how the laser pulse propagates to the QW according to the Maxwell's equation, yielding the Maxwell-SBEs.[9] If the

dielectric environment of the QW yields free-space propagation, the self-consistent driving field follows from[12]

$$E(t) = E_0(t) - \frac{\mu_0\, c}{2n_{\rm QW}} \frac{\partial}{\partial t} \frac{1}{\mathcal{S}} \sum_{\mathbf{k}} d_{\rm eh} P_{\mathbf{k}} \;, \tag{27}$$

where $E_0(t)$ is the temporal dependence of the incoming laser field, μ_0 is the vacuum permeability, c is the speed of light in vacuum, $n_{\rm QW}$ is the refractive index of the QW, and $\mathcal{S}$ is its quantization area. Comparison of Eqs. (17) and (27) indicates that $E_0(t)$ assumes the role of the total atom number $\mathcal{N}$. In other words, the $E(t)$ source of the SBEs and the $N_{\rm C}$ source to the HBEs have an analogous structure where the magnitude of the direct source is self consistently modulated by the excitation level. In addition, the particle–particle interactions renormalize the source similarly, as shown by Eqs. (18) and (26).

As a classification difference of the HBEs and SBEs, each fermion-operator pair increases the cluster number by one whereas each boson operator is counted as a cluster unit. In other words, $P_{\mathbf{k}}$ and $f^{\lambda}_{\mathbf{k}}$ define a complete set of singlets whereas $s_{\mathbf{k}}$ and $f_{\mathbf{k}}$ constitute the doublets. Despite this classification difference, the overall cluster dynamics of HBEs and SBEs is analogous. For example, the interactions couple $P_{\mathbf{k}}$ and $f^{\lambda}_{\mathbf{k}}$ to the doublet correlation due to the hierarchy problem. As derived in Ref. [12], the Coulomb interaction produces a hierarchical coupling

$$i\gamma_{\mathbf{k}} \equiv -\sum_{\mathbf{k},\mathbf{q}} V_{\mathbf{q}} \left(c^{\mathbf{q},\mathbf{k}',\mathbf{k}}_{h,e,e,e} + c^{\mathbf{q},\mathbf{k}',\mathbf{k}}_{h,h,h,e} - \left[c^{\mathbf{q},\mathbf{k}',\mathbf{k}}_{e,e,e,h} + c^{\mathbf{q},\mathbf{k}',\mathbf{k}}_{e,e,e,h} \right]^{\star} \right) \;, \qquad \Gamma^{\lambda}_{\mathbf{k}} \equiv -2\sum_{\mathbf{k},\mathbf{q}} V_{\mathbf{q}}\, {\rm Im} \left[c^{\mathbf{q},\mathbf{k}',\mathbf{k}}_{\lambda,e,e,\lambda} + c^{\mathbf{q},\mathbf{k}',\mathbf{k}}_{\lambda,h,h,\lambda} \right] \;, \tag{28}$$

to the SBEs where $c^{\mathbf{q},\mathbf{k}',\mathbf{k}}_{\lambda,\nu,\nu',\lambda'}$ denote the general class of doublet correlations. As discussed in Supplementary Note 2, one can explain intricate many-body scattering effects by replacing $\gamma_{\mathbf{k}}$ by a diffusive scattering model[12] that includes the central symmetries of the hierarchical coupling; approximation (23) represents an analogous systematic reduction to the hierarchy problem.

Even though the strongly interacting Bose gas and semiconductors share an analogous cluster-dynamics structure, the HBE and SBE approaches are bound to have also fundamental differences because the elementary atom excitations of strongly interacting BEC are bosons while electron–hole excitations of the SBEs are fermions. Most notably, this introduces a couple of sign flips between the HBEs (15)–(17) and SBEs (24); for example, the $\Delta^{\rm ren}_{\mathbf{k}}$ source is multiplied by $(1 + 2f_{\mathbf{k}})$ in Eq. (15) whereas the SBEs contain a Pauli-blocking factor $(-1 + 2f_{\mathbf{k}})$ with $2f_{\mathbf{k}} \equiv f^{e}_{\mathbf{k}} + f^{h}_{\mathbf{k}}$. This meas that the direct source to the transition amplitudes has an opposite sign in the SBEs and HBEs. This seemingly innocent difference changes the equation class so that the SBE excitation satisfies[12] $\left(f_{\mathbf{k}} - \frac{1}{2}\right)^2 + |P_{\mathbf{k}}|^2 \leq \frac{1}{4}$ due to the Pauli exclusion, whereas the HBEs must satisfy Eq. (22) due to the Heisenberg uncertainty relation[11]. Geometrically, the SBE $(f_{\mathbf{k}} - \frac{1}{2}, P_{\mathbf{k}})$ resides within the Bloch sphere[9] whereas the HBE $(f_{\mathbf{k}} + \frac{1}{2}, s_{\mathbf{k}})$ must remain at the upper exterior of a hyperbola. Consequently, Eqs. (15)–(16) are named the *hyperbolic* Bloch equations in Ref. [11]. Similar fermion vs. boson differences also change the specific signs in the Boltzmann scattering induced by triplet scattering, as shown in Refs.[9,12]. On a more subtle level, the HBEs contain more complex energy renormalizations than the SBEs do — especially, the fluctuations of BEC number are absent in the SBEs.

In the SBEs, coupling to a quantized light field[9] can recombine electron–hole pairs via a spontaneous emission of a photon. Analogous light emission through particle annihilation is not relevant in the HBEs. Instead, the HBEs may couple trapped atoms to other hyperfine levels[8], which typically yields atom loss. Besides losses, one can also include electron–hole coupling to lattice vibrations, which dissipates semiconductor energy and transitions as well as brings the system toward the thermodynamic equilibrium. The BEC is thermalized, e.g., via evaporative cooling.

Supplementary Note 3: State dynamics in quantum depletion

As the main predictions of the HBEs, the quantum depletion shows quantum beats and relaxation oscillations as coherent transients, and also a relaxation to a shell state at higher atom densities. These coherent transients are very widely known properties of the SBEs, which makes the explanation of experimentally observed dynamics "trivial" after one has found the connection between the HBEs and SBEs. However, the predicted saturation of quantum depletion to 50% due to shell-state sheltering, is unexpected. This aspect will be explained next.

The homogeneous part of both SBE and HBE coherences identifies an eigenvalue problem whose solutions explain how the individual particles become bound pairs[9,12]. More specifically, the right-hand side of the HBE (15), without the scattering terms, yields the hyperbolic Wannier equation[11]

$$2E^{\rm ren}_{\mathbf{k}} \phi^{R}_{\nu}(\mathbf{k}) + (1 + 2f_{\mathbf{k}}) \sum_{\mathbf{k}'} V_{\mathbf{k}'-\mathbf{k}}\, \phi^{R}_{\nu}(\mathbf{k}') = E_{\nu}\, \phi^{R}_{\nu}(\mathbf{k}) \;, \tag{29}$$

where E_ν is an eigenvalue and $\phi_\nu^R(\mathbf{k})$ is an eigenfunction associated with $s_\mathbf{k}$. For vanishing excitations, $2E_\mathbf{k}^{\text{ren}} = \frac{\hbar^2\mathbf{k}^2}{m}$ is the kinetic energy of an atom pair and the $\sum_{\mathbf{k}'} V_{\mathbf{k}'-\mathbf{k}}$ part describes their mutual interaction. As shown in Ref. [11], the low-density limit of Eq. (29) yields a Schrödinger equation that has the usual series of bound dimers as well as ionized solutions as eigenstates. The dimer state closest to zero energy determines the scattering-length parameterization[11].

The general form of Eq. (29) establishes an asymmetric matrix eigenvalue problem due to the $(1+2f_\mathbf{k})$ factor. Consequently, it has different left-handed and right-handed solutions $\phi_\nu^L(\mathbf{k})$ and $\phi_\nu^R(\mathbf{k})$, respectively. It is straightforward to show that $\phi_\nu^L(\mathbf{k})$ and $\phi_\nu^R(\mathbf{k})$ are connected and normalized[11] by

$$\phi_\nu^L(\mathbf{k}) = \frac{\phi_\nu^R(\mathbf{k})}{1+2f_\mathbf{k}}, \qquad \sum_\mathbf{k} \left[\phi_\lambda^L(\mathbf{k})\right]^\star \phi_\nu^R(\mathbf{k}) = \delta_{\lambda,\nu}\,, \tag{30}$$

respectively. Once all $\phi_\nu^R(\mathbf{k})$ are known, the squeezing amplitude can be converted into the *dimer basis*

$$s_\mathbf{k} \equiv \sum_\nu s_\nu\, \phi_\nu^R(\mathbf{k}) \quad \Leftrightarrow \quad s_\nu = \sum_\mathbf{k} \left[\phi_\nu^L(\mathbf{k})\right]^\star s_\mathbf{k}\,, \tag{31}$$

by applying property (30). For later use, one can express the interaction sum,

$$\sum_{\mathbf{k}'} V_{\mathbf{k}'-\mathbf{k}}\phi_\nu^R(\mathbf{k}') = \left(E_\nu - 2E_\mathbf{k}^{\text{ren}}\right)\, \phi_\nu^L(\mathbf{k})\,, \tag{32}$$

in terms of the $\phi_\nu^L(\mathbf{k})$, after solving the interaction sum from Eq. (29) with the help of the identification (30).

Shell-state sheltering

As shown in the main text, a sufficiently high atom density converts the normal-component to a shell-state that shelters the BEC from further quantum depletion. To explain this phenomenon, one only needs to consider dynamics close to the steady state where the dimer basis can be assumed to be constant in time. In this situation, Eq. (15) can be converted to the dimer basis by projecting its both sides with $\sum_\mathbf{k}\left[\phi_\nu^L(\mathbf{k})\right]^\star$. This straightforward procedure yields

$$i\hbar\frac{\partial}{\partial t}s_\nu = (E_\nu - 2\mu - 2i\gamma)s_\nu + N_\text{C}V_\nu, \qquad V_\nu \equiv \sum_\mathbf{k} V_\mathbf{k}\left[\phi_\nu^R(\mathbf{k})\right]^\star\,. \tag{33}$$

In this dimer picture, it is clear that a fast switch-on of quantum depletion starts to excite *all* dimer as well as ionized states with the weights defined by V_ν. Shortly after the abrupt switch-on, s_ν remains infinitesimal such that only the $N_\text{C}\,V_\nu$ efficiently drives s_ν. This initially excites a broad band of dimer states because Eq. (33) has the same structure as long as $(E_\nu - 2\mu - 2i\gamma)s_\nu$ can be ignored. Once appreciable s_ν builds up, $(E_\nu - 2\mu - 2i\gamma)s_\nu$ contributes and starts to enforce the time-energy uncertainty, which explains the momentum-dependent equilibration of the Tan-contact tail in Supplementary Figure 6 and Fig. 4. As a result, excitation of dimer states with the smallest $|E_\nu - 2\mu|$ becomes favorable, producing a coherent transition from broad-band to narrow-band excitations. This coherent transient explains why the corresponding $f_\mathbf{k}$ evolves from a broad to a narrower shape, as in Figs. 2, 5 and Supplementary Figure 6.

By using property (32), the interaction source becomes $V_\nu = (E_\nu - 2E_0^{\text{ren}})\left[\phi_\nu^L(0)\right]^\star$ where E_0^{ren} defines the energy renormalization at $\mathbf{k}=0$. With the help of this, the steady-state solution of Eq. (33) yields

$$s_\nu = -\frac{N_\text{C}\,V_\nu}{E_\nu - 2\mu - 2i\gamma} = -N_\text{C}\frac{E_\nu - 2E_0^{\text{ren}}}{E_\nu - 2\mu - 2i\gamma}\left[\phi_\nu^L(0)\right]^\star \to -N_\text{C}\frac{E_\nu - 2E_0^{\text{ren}}}{E_\nu - 2\mu}\left[\phi_\nu^L(0)\right]^\star\,. \tag{34}$$

The Lorentzian contribution implies that increasing μ shifts a resonantly excited state to a higher energy. In other words, when the excitation-induced shift (EIS) increases μ, a potential shell state is expected to peak at a higher momentum. This trend explains why the shell-state formation is supported by the EIS, as observed in Fig. 5 of the main text.

Since the measurement has a very small γ, it can be ignored in s_ν, as is done in the last step. As the excitation approaches equilibrium, typically one dimer state $\nu = 1$ dominates the quantum depletion and E_0^{ren} approaches μ, which makes the fraction of Eq. (34) one. With these considerations, the dimer expansion (31) approaches

$$s_\mathbf{k} \to s_1\phi_1^R(\mathbf{k}) \to -N_\text{C}\left[\phi_1^L(0)\right]^\star \phi_1^R(\mathbf{k})\,. \tag{35}$$

In other words, the transition amplitude is directly proportional to the BEC-atom number.

When a shell-state is prominent, $\phi_1^R(\mathbf{k})$ peaks at a nonzero $\mathbf{k}_0$ and N_C becomes large, which makes the peak $s_\mathbf{k}$ large as well. At the same time, the atom density and transition amplitude are connected with the minimum-uncertainty relation (22) (with equality) that has only one physically relevant root, $f_\mathbf{k} = \frac{1}{2}\left(\sqrt{1+4|s_\mathbf{k}|^2}-1\right)$. In the vicinity of the shell-state peak, $\mathbf{k} \in$ peak, $|s_\mathbf{k}|^2$ is very large such that it dominates the square root, yielding

$$f_\mathbf{k} \to |s_\mathbf{k}| \to N_\mathrm{C} \left| \left[\phi_1^L(0)\right]^\star \phi_1^R(\mathbf{k}) \right| \to N_\mathrm{C} \left[\phi_1^L(\mathbf{k})\right]^\star \phi_1^R(\mathbf{k}) \,, \qquad \mathbf{k} \in \text{peak} \,, \tag{36}$$

after property (35) has been applied and $\phi_1^L(0)$ has been replaced by $\phi_1^L(\mathbf{k})$, which is a good approximation close to the shell-state peak. Interestingly, also $f_\mathbf{k}$ scales with N_C in the vicinity of the shell-state peak. One can now compute the total number of normal-component atoms, $N_\mathrm{N} \equiv \sum_\mathbf{k} f_\mathbf{k} \to \sum_{\mathbf{k}\in\text{peak}} f_\mathbf{k}$ that gets its main contribution from the assumed shell-state peak. By inserting limit (36) into this expression, the normal-component atom number becomes

$$N_\mathrm{N} \to N_\mathrm{C} \sum_{\mathbf{k}\in\text{peak}} \left[\phi_1^L(\mathbf{k})\right]^\star \phi_1^R(\mathbf{k}) \to N_\mathrm{C} \,, \tag{37}$$

due to norm (30). The accuracy of the approximations above, e.g. having a pronounced shell-state peak, increases as the atom number is elevated. Therefore, the limit $\mathcal{N} \to \infty$ yields parity $N_\mathrm{N} = N_\mathrm{C}$, which means that the shell state contains 50% of the atoms while 50% of the atoms remain in the BEC. Therefore, increasing the atom number in the trap cannot produce coherent quantum depletion that increases the normal-component fraction F_N above 50% due to shell-state formation. However, before the shell state is fully formed, F_N can transiently exceed 50%, giving rise to relaxation oscillations, as shown in Fig. 3**a** of the main text.

From a liquid to a shell state

The mutual arrangement of atoms can be viewed directly by analyzing the pair correlation function

$$g^{(2)}(\mathbf{r}) \equiv \langle \hat{\Psi}^\dagger(\mathbf{r})\, \hat{\Psi}^\dagger(0)\, \hat{\Psi}(0)\, \hat{\Psi}(\mathbf{r}) \rangle \,, \tag{38}$$

which defines the conditional probability of finding an atom at position $\mathbf{r}$ while another atom is held at the origin. On the field-operator level, $g^{(2)}(\mathbf{r})$ has a similar structure as the atom–atom interaction in Eq. (1). Therefore, one can transform it to the excitation picture with very similar steps as those yielding Eqs. (4) and (9). A straightforward derivation the produces an exact decomposition

$$\begin{aligned} g^{(2)}(\mathbf{r}) \;\equiv\; & \frac{\mathcal{N}(\mathcal{N}-1)}{\mathcal{L}^6} + 2\frac{N_\mathrm{C}\mathcal{N}}{\mathcal{L}^6}\mathrm{Re}\left[n(\mathbf{r})+s(\mathbf{r})\right] + \frac{\mathcal{N}^2}{\mathcal{L}^6}\left(|n(\mathbf{r})|^2+|s(\mathbf{r})|^2\right) \\ & + 4\frac{\sqrt{N_\mathrm{C}}}{\mathcal{L}^6}\sum_{\mathbf{k},\mathbf{k}'}{}' \mathrm{Re}\left[T_{1,2}^{\mathbf{k}',\mathbf{k}}\, e^{i\mathbf{k}\cdot\mathbf{r}}\right] + \sum_{\mathbf{k},\mathbf{k}'}{}' \sum_{\mathbf{q}\neq\mathbf{k},\mathbf{k}'} Q_{2,2}^{\mathbf{q},\mathbf{k}',\mathbf{k}}\, e^{i\mathbf{q}\cdot\mathbf{r}} \,, \end{aligned} \tag{39}$$

where we have identified the doublet contributions

$$f(\mathbf{r}) \equiv \frac{1}{\mathcal{N}}\sum_\mathbf{k} f_\mathbf{k}\, e^{i\mathbf{k}\cdot\mathbf{r}} \,, \qquad s(\mathbf{r}) \equiv \frac{1}{\mathcal{N}}\sum_\mathbf{k} s_\mathbf{k}\, e^{i\mathbf{k}\cdot\mathbf{r}} \quad , \tag{40}$$

normalized by the total atom number $\mathcal{N}$. A system without atom clusters has a constant $g(\mathbf{r}) = \frac{\mathcal{N}(\mathcal{N}-1)}{\mathcal{L}^6}$ that approaches $\frac{\mathcal{N}^2}{\mathcal{L}^6}$ for a large enough atom number. Since $\frac{\mathcal{N}}{\mathcal{L}^3}$ is equal to the atom density ρ, uncorrelated atoms produce $g(\mathbf{r}) = \rho^2$. Whenever the system is dominated by doublet clusters, one may omit triplet and quadruplet contributions; the corresponding normalized pair-correlation function then becomes

$$g(\mathbf{r}) \equiv \frac{g^{(2)}(\mathbf{r})}{\rho^2} = 1 + 2\frac{N_\mathrm{C}}{\mathcal{N}}\,\mathrm{Re}\left[n(\mathbf{r})+s(\mathbf{r})\right] + |n(\mathbf{r})|^2 + |s(\mathbf{r})|^2 \,. \tag{41}$$

For uncorrelated atoms, $g(\mathbf{r})$ is one whereas any genuine $\mathbf{r}$ dependence must stem from the pair-wise correlations.

The quantum-kinetic evolution from a liquid to a shell state is already identified in the $g(r)$ contour-plot of Fig. 4 of the main text. This intriguing dynamics can be studied in more detail by analyzing the time evolution of individual

$r^2(g(r)-1)$ snapshots that determine the deviation from the uncorrelated atom distribution, multiplied by the radial weight. Supplementary Figures 1**a**–1**f** show $r^2(g(r)-1)$ as function of r at six consecutive times t after switching to unitarity. The red line, black line, and shaded area correspond to atom densities of $2.7/\mu\mathrm{m}^3$, $5.5/\mu\mathrm{m}^3$, and $22/\mu\mathrm{m}^3$, respectively; the lowest density was not analyzed in the main text, the intermediate density corresponds to the f-s experiment, and the highest density was shown to evolve toward the shell state in the main text. In all cases, t identifies the evolution time at the unitarity after the fast jump from weak to strong atom–atom interactions.

At $t = 20\,\mu$s (Supplementary Figure 1**a**), all densities produce a very similar $r^2(g(r)-1)$ which shows strong and rapid oscillations up to atom separation $r = 2\,\mu$m. The residual oscillations at larger r result from correlations created already in the weak-interaction regime ($t < 0$). The oscillations form radial shells that have roughly a 220 nm spacing. Such a shell structure is typical for a liquid[34], hence, quantum depletion excites the normal-component to a liquid state — instead of to the gaseous phase. The liquid shells also extend over much larger distances than the typical atom size or atom–atom bond length (220 pm), which shows that quantum depletion creates a liquid phase with long-range order, confirming the conclusions of the main text.

The next time, $t = 50\,\mu$s (Supplementary Figure 1**b**), induces visible deviations between the highest and the two lowest densities. The edge of the liquid shells has now propagated to $r = 5\,\mu$m and the shell spacing grows to 280 nm. In other words, the liquid propagates to longer distances and the shells become more separated. The front edge of the shells has already moved outside the shown range at time $t = 100\,\mu$s (Supplementary Figure 1**c**). The edge is found at $r = 10\,\mu$m and the shell spacing has expanded to 400 nm. The growing shell separation implies that the liquid is not held together by interactions, but it starts to decay making the relative height of the correlated tail smaller. At the same time, the largest ρ result starts to deviate even more from the other densities. This trend continues as t becomes larger; for $t = 200\,\mu$s (Supplementary Figure 1**d**), $t = 400\,\mu$s (Supplementary Figure 1**e**), and $t = 700\,\mu$s (Supplementary Figure 1**f**), only the two lowest densities show the same shell spacing of 510 nm, 840 nm, and 2.4 μm, respectively, as the liquid correlations decay in time.

For the two lowest ρ, the liquid shells almost vanish at $t = 700\,\mu$s because only one full oscillation remains clearly visible, as discussed in the main text. However, the highest ρ shows about three oscillations that stabilize at spacing 2.1 μm which corresponds to the wave length $\lambda = \frac{2\pi}{k_{\mathrm{shell}}} = 2.2\,\mu$m predicted by the shell-state peak wave vector $k_{\mathrm{shell}} = 2.8/\mu$m (Fig. 3**c** in main text). The shape of these stable oscillations is very much present already at $t = 400\,\mu$s, which shows that the liquid has indeed evolved into a stable shell state when ρ becomes high enough. Also the shell state possesses long-range order, even though it does not extend as far as the transient liquid phase, corroborating the conclusions of the main text.

Since the computations include atom momenta over five orders of magnitude, the HBE results also reveal the many-body dynamics at both small and large length scales. With this respect, Supplementary Figure 1 focuses mainly on the large length scale. Supplementary Figure 2 presents the same $r^2(g(r)-1)$ as Supplementary Figure 1, but for a much smaller length scale up to 1.4 nm matching atomic distances. Otherwise, the times and line styles are chosen identically; as the only addition, the vertical dashed line shows the ^{85}Rb bonding length, i.e. $r_0 = 220$ pm, used in the computations.

We observe that the quantum depletion creates $g(\mathbf{r})$ to a very similar shape, on the atomic scale, independent of the excitation. In particular, $g(\mathbf{r})$ decays to zero at the origin due to atom–atom repulsion and peaks at r_0 (vertical dashed line) due to the attractive dip in the atom–atom interactions. The long tail beyond r_0 emerges due to unitarity interaction, which extends the atom–atom attraction to macroscopic scales; the long-range order that follows has already been studied in Supplementary Figure 1. As a general trend, the two lowest densities have a very similar magnitude in $g(\mathbf{r})$ for early times. The overall $g(\mathbf{r})$ decay becomes faster as the density is increased, and for $\rho = 22/\mu\mathrm{m}^3$, $g(r)$ almost vanishes at small distances, which shows that the atom–atom correlations are dominated by the long-range component. This observation is consistent with the shell-state formation.

Supplementary Note 4: On the validity of computations

The general validity of the cluster-expansion approach is already addressed in Supplementary Note 2. such that we may focus on studying how the computed results are affected by the most obvious additional features of the f-s experiment. Therefore, the last part of this supplement is devoted to investigate how the three-body loss and the atom-density profile of the trap effect the results. I will also show explicitly that the HBEs produce gapless excitations, and how the Tan-contact[35] tail of distributions is described by the implemented approach.

Three-body loss and shell state

In the cluster-expansion approach, the three-body loss follows from solving the quantum kinetics of triplets together with the doublets[11]. At the same time, the f-s experiment demonstrates a very weak decay (on a 630μs scale) of the BEC, which is consistent with a reduced formation of clusters beyond doublets (see discussion in Supplementary Note 2). Already the HBEs without three-body loss reproduce the f-s experiment in great detail, cf. Figs. 2, 5 in the main text, and Supplementary Figure 6. Consequently, the approximated HBEs indeed describe the principal effects of quantum-depletion dynamics.

Obviously, the full triplet-quadruplet dynamics[11] includes the complete quantum-kinetic nonequilibrium effects of Efimov trimers/quadmers beyond doublets. Nevertheless, a full quantum-kinetic solution of triplets and quadruplets is very challenging, while the three-body loss is simple to describe with a cubic decay model

$$\frac{d\mathcal{N}}{dt} = -K_3 \mathcal{N}^3 \,, \tag{42}$$

for the total atom number $\mathcal{N}$; a similar approximation has also been identified in Ref. 36. In practice, the loss (42) is added besides the HBEs using a reasonable value for K_3.

The computations are started from the initial atom density $\rho = 22\,\mu\text{m}^3$ as in the shell-state analysis of Fig. 3 in the main text. Supplementary Figure **3a**-i shows how the total atom density evolves in time after a fast switch to unitarity for K_3 producing infinite (black line), $\tau = 12.8\,\text{ms}$ (red line), $\tau = 3.2\,\text{ms}$ (blue line), and $\tau = 800\,\mu\text{s}$ (dashed line), and $\tau = 400\,\mu\text{s}$ (shaded area) decay time at the experimental atom density of $5.5/\mu\text{m}^3$. In other words, this analysis scans the three-body loss through the experimentally observed decay time of $\tau = 630\,\mu\text{s}$. For $\rho = 22\,\mu\text{m}^3$, the density-dependent τ is simply 16 times faster than these numbers due to the quadratic scaling of the "linear" decay constant $K_3 \mathcal{N}^2 \equiv \frac{1}{\tau}$ in Eq. (42).

We observe that the total atom density starts decaying as the system is switched to unitarity at $t = 0$ where the three-body loss starts. The corresponding normal-component fraction F_N is presented in Supplementary Figure **3a**-ii and it initially overshoots above 50% for all cases, followed by relaxation toward 50%. For decay rates faster than 1 ms, the equilibrium fraction is slightly below 50%, which is understandable based on Fig. **3a** in the main text; the large decay simply reduces the total atom number below a level that shelters up to 50% of the BEC.

Supplementary Figure **3b**-i presents the actual normal-component distributions $f_{\mathbf{k}}$ (without the BEC) only $50\,\mu\text{s}$ after the switch on. We conclude that the shell state survives the three-body loss because all cases exhibit an appreciable formation of a peak close to $k = 5\,\mu\text{m}^{-1}$. This is explained by the fast excitation of the normal component and the relatively large atom densities still present at this moment. In other words, the shell state is generated soon after the normal component becomes excited, much before the density decays significantly. The corresponding long-time distributions after $700\,\mu\text{s}$ unitarity evolution are shown in Supplementary Figure **3b**-ii. For a clear comparison, the curves are scaled with the indicated factors. For all three-body loss rates used, the normal component maintains a clear shell structure even when an appreciable amount of three-body loss has elapsed, confirming the robustness of the predicted shell state against the three-body loss.

The visibility of the shell state can be estimated by determining the ratio of the peak height of f_k and its zero-momentum value. For $50\,\mu\text{s}$ after the fast switch, a clear shell-state peak emerges slightly above $k = 5\,\mu\text{m}^{-1}$ and it peaks to 0.8278 (0.3678) while being 0.5755 (0.2086) at $k = 0$ for the computation without (with, $\tau = 400\,\mu\text{s}$) the three-body decay. In other words, the shell-state raises well above the base level of normal-component distribution. In pure numbers, $\tau = \infty$ yields a shell state that peaks 44% above the base whereas a fast $\tau = 400\,\mu\text{s}$ decay produces a 76% peak. Interestingly, the three-body decay makes the shell state relatively more visible at early evolution times. At $t = 700\,\mu\text{s}$, the shell state peaks 134% and 43% above the base for without and with $\tau = 400\,\mu\text{s}$ three-body decay, respectively. Even though the shell state remains clear in both cases, the three-body loss decreases the visibility of the shell state significantly at late times. Yet, even the 43% contrast should be sufficient for a clear shell-state peak at this high density.

Supplementary Figure 3 presents only the normal-component distribution f_k whereas an actual measurement is bound to detect n_k that additionally contains the BEC contribution. If the finite-size effects in detection smear the originally narrow BEC peak to overlap with the shell-state, it is clear the detection of the shell-state becomes more challenging. In the f-s experiment[1], the finite-size effects range roughly up to $k = 3\,\mu\text{m}$, which is sufficiently small to detect the shell state. Furthermore, the k peak of the shell-state shifts with atom density such that density-averaged measurements tend to smear the shell-state peak. Despite these complications, I show in Fig. 5 of the main text that the measured $k^3\,\tilde{n}_k$ contains a peak around $k = 7\,\mu\text{m}^{-1}$ that is consistent with the presence of a weak shell state. For this particular experiment, the shell state is stronger than expected due to the excitation-induced μ shift, which makes shell- state appear at a slightly lower density than predicted with a constant μ computation, as discussed after Eq. (34).

Local-density approximation (LDA)

As a general trend, the trap concentrates atoms toward the minimum of the trap potential, and the shape of the atom distribution can be estimated with the Gross-Pitaevskii equation[2]. For weak interactions and a symmetric parabolic trap, the Thomas-Fermi approximation[2] yields a parabolic density profile as function of radius r,

$$\rho(r) = \rho_0 \left(1 - \tfrac{r^2}{R^2}\right), \quad r < R, \qquad \rho(r) = 0, \quad r \geq R, \tag{43}$$

with the peak density ρ_0 and the atom-cloud radius R. As a result, the total atom number becomes $\mathcal{N}_{\rm tot} = 4\pi \int_0^R dr\, r^2 \rho(r)$ and the probability density of finding an atom at position r is $p(r) = \frac{\rho(r)}{\mathcal{N}_{\rm tot}}$. If a given property $O(\rho)$ is known as function of homogeneous density ρ, its LDA-averaged value follows from

$$\langle O \rangle_{\rm LDA} \equiv 4\pi \int_0^R dr\, r^2\, p(r)\, O(r) = \frac{15}{4\rho_0^2} \int_0^{\rho_0} d\rho\, \rho \sqrt{1 - \tfrac{\rho}{\rho_0}}\, O(\rho), \tag{44}$$

where the integration variables are changed according to relation (43). A straightforward integration produces the average atom density $\langle \rho \rangle = \frac{4}{7}\rho_0$. Since the f-s experiment has only $(6 \pm 9)\%$ of volume change after the jump to unitarity, the $p(r)$ distribution can well be approximated by Eq. (43) also at the regime of strong interactions. In other words, integral (44) provides a good estimate for the density-dependence of the f-s experiment.

In general, the HBE computation allows us to determine $O(\rho)$ at any relevant homogeneous density value. One can then apply the LDA by assuming that the many-body interaction effects contribute locally to the total average (44). The HBEs can then be solved independently at each cell with atom density ρ, yielding a different ρ-dependent HBE-excitation ($s_{\mathbf{k}}(\rho)$, $f_{\mathbf{k}}(\rho)$, $N_{\rm C}(\rho)$) for each cell. In other words, the HBE excitation uniquely defines the local observables $O(\rho)$ as function of ρ. The properties measured then follow straightforwardly from the LDA integral (44). To illustrate the density-profile effects on the coherent quantum depletion, ($s_{\mathbf{k}}(\rho)$, $f_{\mathbf{k}}(\rho)$, $N_{\rm C}(\rho)$) combinations have been solved for 40 densities between zero and $\frac{7}{4}\langle \rho \rangle = \rho_0$. Supplementary Figure 4 compares the LDA averaged normal-component distribution $\langle f_{\mathbf{k}} \rangle_{\rm LDA}$ (solid lines) with a homogeneous-computation $f_{\mathbf{k}}$ (dashed line) at density $\rho = 5.5\mu{\rm m}^{-3}$ for the same snapshot times as shown in Fig. 2**b** of the main text; the average LDA density is set to $\langle \rho \rangle_{\rm LDA} = 5.5\mu{\rm m}^{-3}$. We observe that the LDA averaging modifies the results only slightly at this density range, which confirms that the identified quantum beats, relaxation oscillations, and shell-state formation are robust against the atom-density changes within the trap.

Procedure (44) yields significant averaging only when the excitation-induced effects are included together with the three-body loss (42) because these effects are strongly dependent on the local atom density. Therefore, the excitation-induced study in Fig. 5 of the main text applies averaging (44) over 35 different densities across atom trap.

Gapless excitation spectrum for a shell state

The actual excitation spectrum of the HBEs can be directly analyzed by solving the hyperbolic Wannier equation (29) and Eq. (33) defines which states are directly driven by the BEC. More specifically, the BEC is only resonant with those E_ν states whose $(E_\nu - 2\mu)$ energy difference is vanishing. The inevitable presence of 2γ broadening in Eq. (33) extends the range of significant excitations to concern all those states having $|E_\nu - 2\mu| < 2\gamma$.

To demonstrate explicitly that the HBEs are gapless, Supplementary Figure 5**a** compares the $(E_\nu - 2\mu)$ of a zero-density system (circles) and the final shell state (squares) of Fig. 3**c** (solid line) in the main text. The computed spectrum clearly includes a pair state with a vanishing $(E_\nu - 2\mu)$ in both cases. Consequently, the HBEs indeed provide gapless excitations. In fact, the high-density shell state produces more states within the $|E_\nu - 2\mu| < 2\gamma$ window (shaded rectangle) than the zero-density system, which confirms that the HBEs systematically describe quantum depletion for weakly as well as strongly interacting Bose gases.

As discussed in Supplementary Note 2, the gapless HBE excitations stem from new aspects introduced by the systematic cluster dynamics. From these, the new energy renormalization $\delta\mu$ [Eq. (21)] can diverge if $V_{\mathbf{k}}$ is simply replaced by a constant g, corresponding to the contact-potential implementation; I will call such an implementation the g approach to distinguish it from the $V_{\mathbf{k}}$ approach that is used in my HBE computations to include the full $\mathbf{k}$ dependence of the interaction. The shaded area in Supplementary Figure 5**b** shows the shape of the Morse-potential $V_{\mathbf{k}}$ used in the computations; the corresponding analytic $V_{\mathbf{k}}$ is given e.g. in Ref. 11.

We see that $V_{\mathbf{k}}$ is constant (g) for $|\mathbf{k}| < 10^3/\mu$m, whereas a repulsive peak appears around $|\mathbf{k}| = 10^4/\mu$m. This $V_{\mathbf{k}}$ hosts also a deeply-bound molecular state(s) found at E_1 = -10.6 MHz. Such a state is far from resonant with the BEC driven transitions, which prevents its excitation by quantum depletion. A realistic ^{85}Rb has multiple deeply bound states which remain inert, and I have thoroughly checked that the results do not change if $V_{\mathbf{k}}$ is made deeper

to host more bound molecular states. In other words, only the E_ν structure close to the vanishing energies is relevant for the quantum depletion.

For the g-approach, the large-momentum $s_{\mathbf{k}} \propto \frac{1}{\mathbf{k}^2}$ defines the atom distribution $f_{\mathbf{k}} \to |s_{\mathbf{k}}|^2 \propto \frac{1}{\mathbf{k}^4}$ to have the so-called Tan contact[35] tail. With this scalability, the $\sum_{\mathbf{k}}{}' V_{\mathbf{k}} s_{\mathbf{k}}$ part of Eq. (21) becomes formally diverging for the g implementation. Therefore, one should introduce a renormalization procedure to make the excitation-induced shifts finite. For this purpose, one can either use an extended class of functions[35] or a suitable cut off for the integrals[37].

To see how these renormalization aspects are automatically included in the $V_{\mathbf{k}}$ approach, the solid line in Supplementary Figure 5**b** shows the computed $|\mathbf{k}^2 s_{\mathbf{k}}|$ as function of $\mathbf{k}$ for the shell-state presented in Fig. 3 in the main text. We observe that the $|\mathbf{k}^2 s_{\mathbf{k}}|$ approaches a constant level for $|\mathbf{k}|$ within $10/\mu\text{m}$ and $1000/\mu\text{m}$, corroborating the Tan-contact tail. Once $V_{\mathbf{k}}$ (shaded area) becomes repulsive ($V_{\mathbf{k}} > 0$) and decays above $|\mathbf{k}| = 10^4/\mu\text{m}$, $s_{\mathbf{k}}$ exhibits an oscillatory decay toward zero. As a result, the $V_{\mathbf{k}}$ approach automatically produces a converging $\delta\mu$ and other comparable sums. Therefore, the presented HBE computations differ from analytic implementations on a technical level by describing the atom-atom interaction self-consistently at all length scales.

Supplementary Note 5: Makotyn *et al.* experiment

Contrary to expectations[38,39,40,41], Makotyn *et al.* [1] demonstrated experimentally that a Bose–Einstein condensate (BEC) survives unitarity interactions long enough to exhibit intriguing many-body quantum kinetics. In their experiment, the measured atom-loss rate was slow ($630 \pm 30\,\mu$s) and BEC volume change was small ($6 \pm 9\%$), establishing sufficient stability for systematic many-body studies; more experimental parameters are provided in the main text.

I have chosen conditions and material parameters according to the Makyton *et al.* fast-switch experiment[1] (f-s experiment) in order to identify coherent quantum depletion in BECs. To provide an effortless one-to-one comparison between my computations and the f-s experiment, I have carefully read the f-s experiment data from the inset to Fig. 2 in Ref. [1] and present the measured column distributions in Supplementary Figure 6**a** exactly the same way as Fig. 2**a** in the main text. In numbers, the low momentum $\tilde{n}_k$ builds up in roughly 100μs while the high-momentum $\tilde{n}_k$ saturates significantly faster, in roughly 35μs. Similar buildup times have been predicted in Refs. 36,42. To the best of my knowledge, only the HBE kinetics explains the overall shape of the measured $\tilde{n}_{\mathbf{k}}$, and connects the dynamics to coherently driven atom clusters as well as ultrafast excitations in semiconductors. A quantitative experiment–theory comparison is presented in Fig. 5 of the main text.

The f-s experiment distributions were measured[1] at time t after evolution at unitary using resonant, high-intensity absorption imaging. To record atom distributions, the system was rapidly swept from unitarity back to weak interactions at time t, and the trap was simultaneously opened to allow for ballistic expansion before imaging the cloud of atoms. The atom positions in the expanded cloud then reveal the momentum distribution; three expansion times (7, 13 and 25 ms) were applied in the f-s experiment to increase the dynamic range of data and to reduce the effect of finite BEC size on the imaging. This imaging scheme detected column projection of the actual atom distribution $n_{\mathbf{k}}$, as illustrated in Supplementary Figure 6**a**, yielding the column distribution

$$\tilde{n}_{\mathbf{k}_\parallel} = \mathcal{L}^3 \int_{-\infty}^{\infty} dk_z \, n_{\sqrt{\mathbf{k}_\parallel^2 + k_z^2}} \,, \tag{45}$$

where $\mathcal{L}^3$ is the quantization volume. Due to the spherical symmetry of $n_{\mathbf{k}}$, also $\tilde{n}_{\mathbf{k}_\parallel}$ depends only on the magnitude of $|\mathbf{k}_\parallel|$. Connection (45) reduces to Eq. (2) of the main text when $|\mathbf{k}_\parallel|$ is denoted by k and the quantization volume $\mathcal{L}^3 = \frac{\mathcal{N}}{\langle\rho\rangle}$ is expressed using the initial atom number $\mathcal{N}$ and the average atom density $\langle\rho\rangle$. The explicit density-averaging procedure is discussed in Supplementary Note 4 where I show that the f-s experiment is explained by computations using a homogeneous density ρ instead of $\langle\rho\rangle$. Furthermore, the role of atom loss on coherent quantum depletion is discussed in Supplementary Note 4 by including a three-body loss in the HBEs.

To verify that definition (45) agrees with the experimental $\tilde{n}_k$ calibration, one can check whether Eq. (45) reproduces the norm provided in the caption of Fig. 2 in Ref. [1]. One can start by computing the total atom number

$$\mathcal{N}(t) = \sum_{\mathbf{k}} n_{\mathbf{k}} = \frac{\mathcal{L}^3}{(2\pi)^3} \int d^3k \, n_{\mathbf{k}} = \frac{\mathcal{L}^3}{8\pi^3} \int d^2k_\parallel \int_{-\infty}^{\infty} n_{\sqrt{\mathbf{k}_\parallel^2 + k_z^2}} = \frac{1}{8\pi^3} 2\pi \int_0^\infty dk \, k \, \tilde{n}_k \,, \tag{46}$$

where $\frac{2\pi}{\mathcal{L}} = \Delta k$ is the size of the wave-vector discretization needed to convert the sum into an integral. In the third step, the integration is performed in the cylindrical coordinates, which identifies the column distribution (45); also the rotation symmetry of the distributions is applied here. It is now straightforward to rearrange result (46) into $2\pi \int_0^\infty dk \, k \, \tilde{n}_k = 8\pi^3 \mathcal{N}(t)$ which is exactly the relation provided in the caption to Fig. 2 in Ref. [1]. By using the

experimentally known[1] values $\langle \rho \rangle = 5.5/\mu\text{m}^3$ and $\mathcal{N} = 6 \times 10^4$ in Eq. (2) of the main text, the computed $\tilde{n}_k$ is indeed normalized in the same way as the f-s experiment.

Supplementary References

[1] Makotyn, P., Klauss, C., Goldberger, D., Cornell, E. & Jin, D. *Universal dynamics of a degenerate unitary Bose gas. Nature Phys.* **10**, 116–119 (2014).

[2] Dalfovo, F., Giorgini, S., Pitaevskii, L. & Stringari, S. Theory of Bose–Einstein condensation in trapped gases. *Rev. Mod. Phys.* **71**, 463–512 (1999).

[3] Andersen, J. O. Theory of the weakly interacting Bose gas. *Rev. Mod. Phys.* **76**, 599–639 (2004).

[4] Leggett, A. J. Bose–Einstein condensation in the alkali gases: Some fundamental concepts. *Rev. Mod. Phys.* **73**, 307–356 (2001).

[5] Proukakis, N. & Jackson, B. Finite-temperature models of Bose–Einstein condensation. *Journal of Physics B: Atomic, Molecular and Optical Physics* **41**, 203002 (2008).

[6] Blakie, P., Bradley, A., Davis, M., Ballagh, R. & Gardiner, C. Dynamics and statistical mechanics of ultra-cold Bose gases using c-field techniques. *Adv. Phys.* **57**, 363–455 (2008).

[7] Dalibard, J., Gerbier, F., Juzeliūnas, G. & Öhberg, P. Colloquium: Artificial gauge potentials for neutral atoms. *Rev. Mod. Phys.* **83**, 1523–1543 (2011).

[8] Kira, M. Excitation picture of an interacting Bose gas. *Annals of Physics* **351**, 200 – 249 (2014).

[9] Kira, M. & Koch, S. W. *Semiconductor Quantum Optics* (Cambridge University Press, 2011), 1. edn.

[10] Mootz, M., Kira, M. & Koch, S. W. Sequential build-up of quantum-optical correlations. *J. Opt. Soc. Am. B* **29**, A17–A24 (2012).

[11] Kira, M. Hyperbolic Bloch equations: atom-cluster kinetics of an interacting Bose gas. Preprint at http://arxiv.org/abs/1407.4927 (2014).

[12] Kira, M. & Koch, S. W. Many-body correlations and excitonic effects in semiconductor spectroscopy. *Prog. Quantum Electron* **30**, 155 – 296 (2006).

[13] Wick, G. C. The evaluation of the collision matrix. *Phys. Rev.* **80**, 268–272 (1950).

[14] Kira, M. & Koch, S. W. Cluster-expansion representation in quantum optics. *Phys. Rev. A* **78**, 022102 (2008).

[15] Walls, D. F. & Milburn, G. J. *Quantum Optics* (Springer-Verlag, New York, 2008), 2. edn.

[16] Emsley, J. *The Elements* (Oxford Univ. Press, 1998), 3. edn.

[17] Cordero, B. *et al.* Covalent radii revisited. *Dalton Trans.* 2832–2838 (2008).

[18] Morse, P. M. Diatomic molecules according to the wave mechanics. ii. vibrational levels. *Phys. Rev.* **34**, 57–64 (1929).

[19] Baranger, M. Self-consistent field theory of nuclear shapes. *Phys. Rev.* **122**, 992–996 (1961).

[20] Goodman, A. Self-consistent symmetries of the Hartree–Fock–Bogoliubov equations in a rotating frame. *Nuclear Physics A* **230**, 466 – 476 (1974).

[21] Mang, H. The self-consistent single-particle model in nuclear physics. *Physics Reports* **18**, 325 – 368 (1975).

[22] Zaremba, E., Nikuni, T. & Griffin, A. Dynamics of trapped Bose gases at finite temperatures. *Journal of Low Temperature Physics* **116**, 277–345 (1999).

[23] Bender, M., Heenen, P.-H. & Reinhard, P.-G. Self-consistent mean-field models for nuclear structure. *Rev. Mod. Phys.* **75**, 121–180 (2003).

[24] Milstein, J., Menotti, C. & Holland, M. Feshbach resonances and collapsing Bose–Einstein condensates. *New Journal of Physics* **5**, 52 (2003).

[25] Wüster, S., Hope, J. J. & Savage, C. M. Collapsing Bose–Einstein condensates beyond the Gross-Pitaevskii approximation. *Phys. Rev. A* **71**, 033604 (2005).

[26] Griffin, A. Conserving and gapless approximations for an inhomogeneous Bose gas at finite temperatures. *Phys. Rev. B* **53**, 9341–9347 (1996).

[27] Shacklette, J. M. & Cundiff, S. T. Role of excitation-induced shift in the coherent optical response of semiconductors. *Phys. Rev. B* **66**, 045309 (2002).

[28] Wang, H. *et al.* Transient nonlinear optical response from excitation induced dephasing in gaas. *Phys. Rev. Lett.* **71**, 1261–1264 (1993).

[29] Lindberg, M. & Koch, S. W. Effective Bloch equations for semiconductors. *Phys. Rev. B* **38**, 3342–3350 (1988).

[30] Haug, H. & Koch, S. *Quantum Theory of the Optical and Electronic Properties of Semiconductors (5th Edition)* (World Scientific, 2009).

[31] Khitrova, G., Gibbs, H. M., Jahnke, F., Kira, M. & Koch, S. W. Nonlinear optics of normal-mode-coupling semiconductor microcavities. *Rev. Mod. Phys.* **71**, 1591–1639 (1999).

[32] Hofmann, M. *et al.* Gain spectra of (GaIn)(NAs) laser diodes for the 1.3-m-wavelength regime. *Applied Physics Letters* **78**, 3009–3011 (2001).

[33] Smith, R. P. *et al.* Extraction of many-body configurations from nonlinear absorption in semiconductor quantum wells. *Phys. Rev. Lett.* **104**, 247401 (2010).

[34] Jorgensen, W. L., Chandrasekhar, J., Madura, J. D., Impey, R. W. & Klein, M. L. Comparison of simple potential

functions for simulating liquid water. *The Journal of Chemical Physics* **79**, 926–935 (1983).

[35] Tan, S. Energetics of a strongly correlated Fermi gas. *Annals of Physics* **323**, 2952 – 2970 (2008).

[36] Sykes, A. G. *et al.* Quenching to unitarity: Quantum dynamics in a three-dimensional Bose gas. *Phys. Rev. A* **89**, 021601 (2014).

[37] Kokkelmans, S. J. J. M. F., Milstein, J. N., Chiofalo, M. L., Walser, R. & Holland, M. J. Resonance superfluidity: Renormalization of resonance scattering theory. *Phys. Rev. A* **65**, 053617 (2002).

[38] Papp, S. B. *et al.* Bragg Spectroscopy of a Strongly Interacting Rb^{85} Bose–Einstein Condensate. *Phys. Rev. Lett.* **101**, 135301 (2008).

[39] Navon, N. *et al.* Dynamics and thermodynamics of the low-temperature strongly interacting Bose gas. *Phys. Rev. Lett.* **107**, 135301 (2011).

[40] Smith, R. P., Beattie, S., Moulder, S., Campbell, R. L. D. & Hadzibabic, Z. Condensation dynamics in a quantum-quenched Bose gas. *Phys. Rev. Lett.* **109**, 105301 (2012).

[41] Wild, R. J., Makotyn, P., Pino, J. M., Cornell, E. A. & Jin, D. S. Measurements of tan's contact in an atomic Bose–Einstein condensate. *Phys. Rev. Lett.* **108**, 145305 (2012).

[42] Rançon, A. & Levin, K. Equilibrating dynamics in quenched Bose gases: Characterizing multiple time regimes. *Phys. Rev. A* **90**, 021602 (2014).
